# Tracing Sub-Structure in the European American Population with PCA-Informative Markers

**DOI:** 10.1371/journal.pgen.1000114

**Published:** 2008-07-04

**Authors:** Peristera Paschou, Petros Drineas, Jamey Lewis, Caroline M. Nievergelt, Deborah A. Nickerson, Joshua D. Smith, Paul M. Ridker, Daniel I. Chasman, Ronald M. Krauss, Elad Ziv

**Affiliations:** 1 Department of Molecular Biology and Genetics, Democritus University of Thrace, Alexandroupoli, Greece; 2 Department of Computer Science, Rensselaer Polytechnic Institute, Troy, New York, United States of America; 3 Department of Molecular and Experimental Medicine, Scripps Genomic Medicine, The Scripps Research Institute, La Jolla, California, United States of America; 4 Department of Psychiatry, University of California at San Diego, La Jolla, California, United States of America; 5 Department of Genome Sciences, University of Washington, Seattle, Washington, United States of America; 6 Center for Cardiovascular Disease Prevention, Divisions of Cardiovascular Diseases and Preventive Medicine, Brigham and Women's Hospital, Boston, Massachusetts, United States of America; 7 Division of Preventive Medicine, Brigham and Women's Hospital, Boston, Massachusetts, United States of America; 8 Children's Hospital Oakland Research Institute, Oakland, California, United States of America; 9 Division of General Internal Medicine, Institute for Human Genetics, University of California San Francisco, San Francisco, California, United States of America; 10 Comprehensive Cancer Center, University of California San Francisco, San Francisco, California, United States of America; University of Chicago, United States of America

## Abstract

Genetic structure in the European American population reflects waves of migration and recent gene flow among different populations. This complex structure can introduce bias in genetic association studies. Using Principal Components Analysis (PCA), we analyze the structure of two independent European American datasets (1,521 individuals–307,315 autosomal SNPs). Individual variation lies across a continuum with some individuals showing high degrees of admixture with non-European populations, as demonstrated through joint analysis with HapMap data. The CEPH Europeans only represent a small fraction of the variation encountered in the larger European American datasets we studied. We interpret the first eigenvector of this data as correlated with ancestry, and we apply an algorithm that we have previously described to select PCA-informative markers (PCAIMs) that can reproduce this structure. Importantly, we develop a novel method that can remove redundancy from the selected SNP panels and show that we can effectively remove correlated markers, thus increasing genotyping savings. Only 150–200 PCAIMs suffice to accurately predict fine structure in European American datasets, as identified by PCA. Simulating association studies, we couple our method with a PCA-based stratification correction tool and demonstrate that a small number of PCAIMs can efficiently remove false correlations with almost no loss in power. The structure informative SNPs that we propose are an important resource for genetic association studies of European Americans. Furthermore, our redundancy removal algorithm can be applied on sets of ancestry informative markers selected with any method in order to select the most uncorrelated SNPs, and significantly decreases genotyping costs.

## Author Summary

Genetic association studies search to identify disease susceptibility genes through the analysis of genetic markers such as single nucleotide polymorphisms (SNPs) in large numbers of cases and controls. In such settings, the existence of sub-structure in the population under study (i.e. differences in ancestry among cases and controls) may lead to spurious results. It is therefore imperative to control for this possible bias. Such biases may arise for example when studying the European American population, which consists of individuals of diverse ancestry proportions from different European countries and to some degree also from African and Native American populations. Here, we study the genetic sub-structure of the European American population, analyzing 1,521 individuals for over 300,000 SNPs across the entire genome. Applying a powerful method that is based on dimensionality reduction (Principal Components Analysis), we are able to identify 200 SNPs that successfully represent the complete dataset. Importantly, we introduce a novel method that effectively removes redundancy from any set of genetic markers, and may prove extremely useful in a variety of different research scenarios, in order to significantly reduce the cost of a study.

## Introduction

The first Europeans from the Old World to land in what is now US territory were Columbus' men in 1493. The initial colonization of the region by the Spanish, English, Scots and Irish, French, Dutch, Swedes, Germans, Italians and Portuguese during the 16th and 17th centuries was followed in the 19th and early 20th century by waves of millions of newcomers originating from the northwestern to the southeastern corners of Europe [1]. Thus, the present day European American population is a mosaic of people that represent different levels of admixture between diverse European populations and, to some degree, also with Native American and African American populations.

The identification of population genetic structure has been discussed at length in recent literature, due to the potential bias it can introduce in association studies, searching for susceptibility genes for common complex disorders [2]–[5]. Population stratification is a source of confounding in case-control studies, when allele-frequency heterogeneity that is unrelated with the studied phenotype is coupled with disease-risk heterogeneity and biased sampling in cases and controls. Although European populations were initially considered genetically quite homogeneous, it has recently been shown that significant patterns of structure within Europe along a north to south axis do exist and that unidentified population stratification in European derived populations (European Americans) can lead to spurious associations with disease [5]–[8].

As genotyping of thousands of individuals for hundreds of thousands of markers becomes feasible [9]–[14], and genome wide association studies in large samples of European American populations become increasingly common [15], identifying and correcting for population stratification will undoubtedly play a central part in the quest to unravel the genetic basis of complex traits. The uniform adjustment proposed by the method of genomic control could be too conservative [16],[17], while structured association testing is computationally impractical for very large datasets [18]. Price et al. [19] have shown that Principal Components Analysis (PCA), a powerful linear dimensionality reduction technique can be used as a computationally efficient tool to correct for stratification in the setting of genome wide association studies without loss in power.

Identifying a small set of markers that could be used for inference of population structure and adjustment for stratification is of particular importance in order to reduce genotyping costs in studies seeking to replicate the findings of large-scale genome-wide projects or when pursuing specific loci as candidate susceptibility genes. Most existing metrics to select ancestry informative markers (AIMs) are allele frequency based and demand prior knowledge of the ancestry of the studied individuals. Consequently, measures like *F_st_*, *δ* and informativeness for assignment [20]–[25], require prior assumptions about individual ancestry and cannot be directly applied to admixed populations, like European Americans, in order to identify a panel of genetic markers that can reproduce the structure of the dataset. European American AIMs had so far been proposed in two recent large studies targeting distinct European populations, used as proxies for European American ancestry [7],[8]. Our work here as well as two studies parallel to ours described in [26],[27] are the first to attempt the identification of structure informative SNPs through the direct analysis of genomewide datasets of European Americans. All three of these studies are PCA-based. However, here, we directly leverage the power of PCA for the selection of AIMs [28], without the need for any intermediate steps, such as assigning individuals to clusters, in order to use allele frequency based metrics.

We have recently introduced an unsupervised method for the selection of ancestry and structure informative SNPs (PCA-correlated SNPs or PCA-informative SNPs-PCAIMs) [28]. Our method does not require prior hypotheses or knowledge of individual ancestry and thus is well-suited for selecting AIMs in admixed populations. In this paper, we employ it to analyze a dense, genome-wide dataset (approx. 307,000 SNPs) of more than 1,500 European Americans from two different studies [29],[30]. Our main goal is the identification of a small panel of structure informative SNPs in the European American population. The contributions of this paper are three-fold. *First*, from a statistical perspective, we propose a methodology to remove redundancy from any set of genetic markers, an issue that arises with all existing methods (supervised or unsupervised) for the selection of ancestry or structure informative markers, since the “scoring” of the SNPs in all of these methods does not take into account any correlation between them. We reduce the redundancy removal problem to a well-known problem in numerical linear algebra, the so-called Column Subset Selection Problem [31] and we propose an efficient and accurate algorithm that filters out redundant SNPs. *Second*, we demonstrate that as few as 200 SNPs selected with our methodology can be used to very accurately predict the fine structure of European Americans as identified by PCA, and we employ cross-validation experiments to verify the accuracy of our approach. *Third*, we show that our method can be coupled with PCA-based stratification correction tools (such as EIGENSTRAT [19]) for accurate stratification correction with significant genotyping savings. Using simulated data we experimentally demonstrate that 100–200 PCAIMs can be used to correct for stratification while maintaining power in association studies.

## Methods

### Datasets

We studied two independent European American datasets. The first dataset (CHORI dataset-Children's Hospital Oakland Research Institute), consists of 980 individuals, that were collected as part of two community-based clinical trials evaluating the anti-inflammatory effects of statins. 305 of these samples (part of the CAP study [32]) were collected from the San Francisco Bay Area and Los Angeles. These individuals all had to report at least 3 grandparents of European or Caucasian background. Another 675 individuals were part of a clinical trial that included a large number of sites across the U.S. (PRINCE study [33]). These individuals were self-reported white or Caucasian but no additional information was collected about their parents. All 980 individuals were genotyped using the Illumina Infinium 310K array in one laboratory under the same conditions. The second dataset that we studied here (CORIELL dataset), is a publicly available dataset that has been previously described [29], and consists of the same SNPs genotyped for 541 samples (data available from the SNP Resource at the NINDS Human Genetics Resource Center DNA and Cell Line Repository (http://ccr.coriell.org/ninds/). These are samples from patients with Parkinson's disease and neurologically normal controls, curated at the Coriell institute. Again, genotyping was performed using the Illumina platform (in the laboratory of Drs. Singleton and Hardy (NIA, LNG), Bethesda, MD USA). For all datasets we only considered genotypes for SNPs on autosomal chromosomes in our analysis. Finally, as a third dataset, we also studied the same SNPs using data available from the HapMap database on the HapMap Yoruba (YRI), CEPH European (CEU), Chinese (CHB), and Japanese (JPT) samples [34],[35].

### Preprocessing and Encoding the Data

The proportion of missing entries in the above datasets was very small (on average less than 0.1%). As a quality control step, we excluded all SNPs with more than 5% missing entries (separately on each of the three datasets). This step further reduced the number of missing entries to less that 0.07% on average. We also excluded from the analysis a small number of SNPs that were not in Hardy-Weinberg equilibrium (HWE). After these preprocessing steps we were left with a total of 307,315 autosomal SNPs that all three datasets had in common.

In order to simplify and speed up our computations, we filled in the (very small) number of missing entries randomly so that HWE is satisfied for each SNP. The probabilistic filling in was performed separately for each dataset, and separately in each population of the HapMap data. We then transformed the raw data to numeric values, without any loss of information, in order to apply our linear algebraic methods. Consider a dataset of a population *X* consisting of *m* subjects and assume that for each subject *n* biallelic SNPs have been assayed. Thus, we are given a table *T^x^*, consisting of *m* rows and *n* columns. Each entry in the table is a pair of bases, ordered alphabetically. We transform this initial data table to an integer matrix *A^x^* which consists of *m* rows (one for each subject), and *n* columns (one for each SNP). Each entry of *A^x^* will be −1, 0, +1, or empty. Let *B*_1_ and *B*_2_ be the bases that appear in the *j*-th SNP (in alphabetical order). If the genotypic information for the *j*-th SNP of the *i*-th individual is *B*_1_ *B*_1_ the (*i,j*) -th entry of *A^x^* is set to +1; else if it is *B*_1_ *B*_2_ the (*i,j*)-th entry of *A^x^* is set to 0; else if it is *B*_2_ *B*_2_ the (*i,j*)-th entry of *A^x^* is set to −1 [28],[36].

### The Singular Value Decomposition and Outlier Removal

We carefully studied the two European American datasets for outlier individuals. In the CHORI dataset, we identified five pairs of individuals that showed a very high degree of allele sharing and removed these ten subjects from all further analysis. In particular, we determined the proportion of allele sharing between all pairs of individuals for 1000 randomly selected markers, approximately equally spaced throughout the genome, and subtracted it from the proportion of allele sharing expected under a randomly mating population with the same allele frequencies. These five pairs included one pair that had 100% sharing for all 1000 markers (indicating either an identical twin or a duplicate sample) and four others that had significantly higher than expected excess allele sharing, suggesting that they were related.

We subsequently used Principal Components Analysis and the Singular Value Decomposition to detect outliers. In particular, given *m* subjects and *n* SNPs, let the *m*×*n* matrix *A* denote the subject-SNP matrix encoded as described above. After mean-centering the columns (SNP genotypes) of *A*, the SVD of the matrix returns *m* pairwise orthonormal vectors *u^i^*, *n* pairwise orthonormal vectors *v^i^*, and *m* non-negative singular values *σ_i_* such that *σ*_1_≥*σ*_2_≥…≥*σ_m_*≥0. The matrix *A* may be written as a sum of outer products as
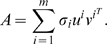
(1)


Each triplet (*σ_i_*,*u^i^*,*ν^i^*) may be used to form a principal component of *A*. Formally, the *i*-th most significant principal component of a matrix *A* is the rank-one matrix that is equal to 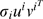. In our setting, the left singular vectors (the *u^i^* 's) are linear combinations of the columns (SNPs) of *A* and will be called eigenSNPs [37]. Notice that a principal component is a matrix, whereas an eigenSNP is just a column vector. PCA is a well-known dimensionality reduction technique that, in this case, represents all subjects with respect to a small number of eigenSNPs, corresponding to the top few principal components. All further analysis is then performed on this low-dimensional representation.

Figure S1 shows the plot of the 970 CHORI individuals, the 541 CORIELL individuals, and the HapMap European, African and Asian samples, projected on their top three eigenSNPs (as we shall argue in Results the top eigenSNP is the most informative). This plot illustrates how a few subjects from our European American datasets are “pulled” towards the African and Asian HapMap populations. Based on this analysis, we discarded 12 individuals from the CHORI dataset and 2 individuals from the CORIELL dataset that were far from the vast majority of the European American subjects and seem to have a higher degree of non-European ancestry (Figure S1). Overall, out of the 1521 subjects in the CHORI and CORIELL datasets, we discarded a total of 24 subjects (ten suspiciously similar subjects and 14 outliers). Thus, we were left with 1497 subjects of European American ancestry, genotyped for 307,315 SNPs.

### Selecting PCAIMs and Removing Redundancy

In order to select ancestry informative markers, we used the procedure that we described previously [28], [38]–[40]. This procedure is based on the well-documented fact that Principal Components Analysis reveals population structure. More specifically, a number of studies have verified that retaining the top few eigenSNPs in datasets that contain individuals from a number of different populations, or even admixed populations, efficiently reveals the ancestry of the individuals [19], [28], [41]–[44]. The PCAIM selection algorithm first determines the number of significant principal components (and thus the number of informative eigenSNPs) in the data, and then assigns a score to each SNP. Higher scores correspond to SNPs that correlate well with all informative eigenSNPs. The algorithm returns the top scoring SNPs, and we have demonstrated that these PCAIMs are very efficient for ancestry prediction [28].

This algorithm does not take any special measures in order to avoid redundancy in the set of identified markers. As we will also discuss later here, redundancy may arise in sets of AIMs selected with any of the existing methods (eg. *δ*, *F_st_*, informativeness, PCAIMs). Redundancy in the case of dense sets of SNP markers is due to tight linkage disequilibrium. Given the increased marker density in the genomewide datasets that are becoming available today, this may lead to significant loss in efficiency by selecting highly correlated markers. It is therefore important to add a redundancy removal step after the initial selection of structure informative markers.

We propose a simple, efficient methodology to deal with this issue. Our methodology is based on reducing the redundancy removal problem to the so-called Column Subset Selection Problem. The latter problem is well studied in the Numerical Linear Algebra literature, and many algorithms, with various accuracy vs speed tradeoffs, have been proposed [45]. More specifically, assume that the top *r*≪*n* highest scoring SNPs are retained as PCAIMs. Thus, we are given a matrix *Ã* that has *m* rows (one for each subject) but only *r* columns (one for each PCAIM). Recall that *n* is the total number of SNPs, and could be in the order of hundreds of thousands, whereas we expect *r* to be in the order of thousands. Our goal is to only retain a small number (say *k*) of columns of *Ã* that are as uncorrelated as possible. A naive way of solving this problem would be to examine all 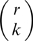 possible choices of sets of *k* SNPs and keep a set that has no pairs of highly correlated SNPs. This is computationally infeasible even for very small values of *k* (say ten) if *r* is even a thousand. Consider the following definition for the Column Subset Selection Problem (CSSP):

**Definition 1:** *Given an m×r matrix Ã and a positive integer k, pick k columns (SNPs) of Ã such that the maximal Pearson correlation coefficient between all* 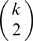 *pairs of the selected columns (SNPs) is minimized.*

In words, recall that a large (close to one) Pearson correlation coefficient between a pair of SNPs would imply that one of the two SNPs in redundant. Thus, the above problem formulation seeks to minimize the maximal correlation between any pair of selected SNPs, and thus ensure that limited or no redundancy exists. Even though solving the above optimization problem exactly is hard, efficient approximation algorithms exist. For the purposes of this paper, we chose to use an algorithm called *greedy QR*, that was proposed by Golub in [31] and was subsequently analyzed by Gu and Eisenstat in [46]. The algorithm essentially works in *k* iterations; in the first iteration, the first column of *Ã* (the top PCAIM) is picked; in the second iteration, a column of *Ã* is picked that is as uncorrelated with the first column as possible; in the third iteration the chosen column has to be as uncorrelated as possible with the first two columns, etc. When expressed in linear algebraic notation, this iterative procedure boils down to a permuted *QR* decomposition of a matrix, and can be performed efficiently. In particular, an efficient implementation of this algorithm is available in MatLab, and runs in less than one minute when *r* is in the order of thousands and any value of *k* less than *r*.

### Simulated Association Studies

In order to illustrate the potential of the proposed PCAIMs for the correction of stratification in association studies, we run a large simulated association study that closely followed the simulated association study in Price et al. [19]. More specifically, Price et al. [19] demonstrated how EIGENSTRAT (a PCA-based procedure) could efficiently identify population structure and remove stratification from association studies on populations with similar structural characteristics with the European American population.

To demonstrate the performance of PCAIMs to correct for stratification in association studies on admixed populations with similar characteristics with European American populations, we followed the methods of [19] to generate an admixed population of 1,000 individuals genotyped on 100,000 SNPs (see Text S1 for details). Thus, we created a 1,000×100,000 matrix *A* of genotypes. We then estimated the number of significant principal components, both by looking at the singular values, as well as by the permutation test of [28]. As we will discuss in the Results section, one eigenSNP was deemed significant and was interpreted as ancestry. We then picked panels of PCAIMs from the 100,000 SNPs in order to predict the ancestry of the 1,000 subjects.

We created large sets of random, stratified, and causal SNPs (100,000 SNPs in each case) following the methods described by Price et al. [19] (see Text S1). We performed ten repetitions, and generated sets of 100,000, since we did not observe any change in the fourth decimal digit of the reported results by increasing the set size to 1,000,000. Affection status for individuals in the admixed population was determined randomly according to an “ancestry risk” parameter *r* as defined previously [19]. Results are reported for both *r* = 2 and 3.

Correlation with affection status was determined by taking the Armitage trend statistic of each SNP with the affection status, with the significance threshold set to 10^−4^. For comparison purposes we chose the same threshold as in [19]. Correction for ancestry was first performed using the algorithm of EIGENSTRAT and looking at the top ten eigenSNPs of the full SNP-subject matrix (mean centering was performed). We then performed correction for stratification by looking at the first eigenSNP of the matrix consisting of the panel of selected PCAIMs. Adjustment of genotypes essentially corresponds to “projecting out” the component of each SNP that lies in the subspace spanned by the ancestry prediction. After performing this simple linear algebraic operation on every SNP, the Armitage trend statistic was re-run on the residual of each SNP.

## Results

### Population Substructure and Ancestry in European Americans

**Figure 1:**
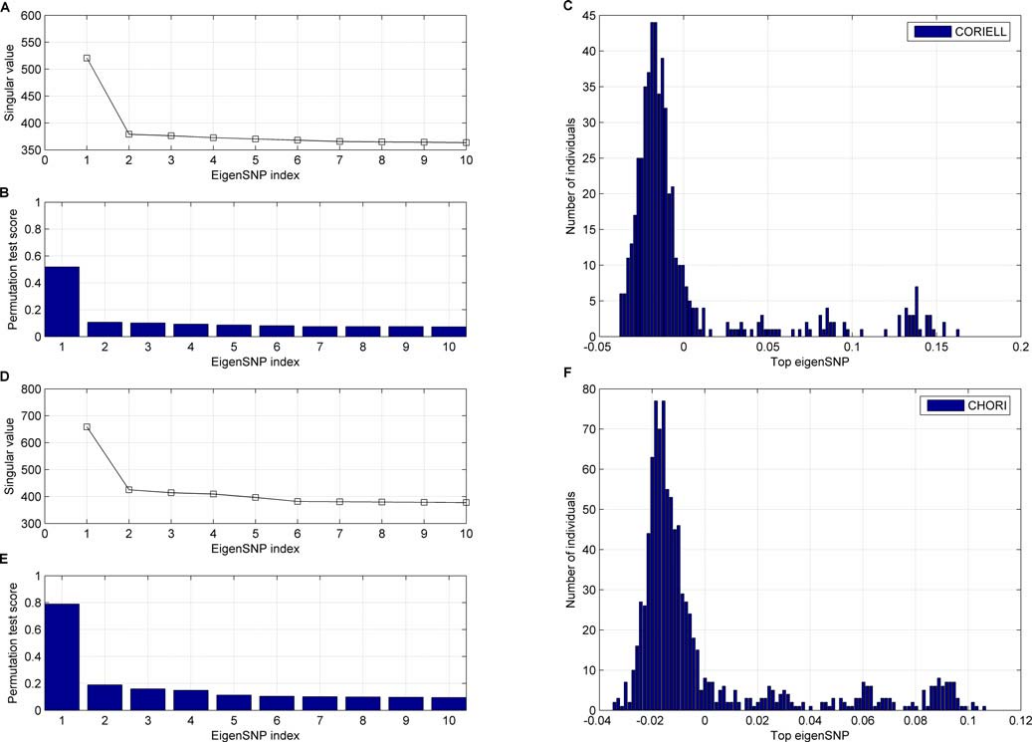
Singular values and test of significance of principal components in the CHORI and CORIELL datasets. (A) Histogram of the first eigenSNP of the CORIELL dataset. (B) The singular values corresponding to the first up to the tenth eigenSNP of the CORIELL dataset. (C) The results of the permutation test to determine the significance of the principal components of the CORIELL dataset. Higher values on the *y*-axis correspond to principal components containing significantly more structure than a random component would. (D) Histogram of the first eigenSNP of the CHORI dataset. (E) The singular values corresponding to the first up to the tenth eigenSNP of the CHORI dataset. (F) The results of the permutation test to determine the significance of the principal components of the CHORI dataset.

We first examined the number of significant principal components in the two European American datasets that we studied (CHORI and CORIELL). Figure 1 (panel A) shows the top few singular values of the CORIELL subject-SNP matrix, and Figure 1 (panel D) shows the top few singular values of the CHORI subject-SNP matrix. Clearly, there is a significant gap between the first singular value and the remaining ones in both cases. This is a strong indication that the top principal component is the most informative in both datasets and suggests that subsequent principal components may not be of interest. To further validate this finding, we ran the permutation test that we have recently described [28]. This permutation test essentially measures the ratio of “information” that the *i*-th principal component contains when compared to the amount of structure in a random matrix. When this ratio is sufficiently high, the principal component is deemed as informative. Again, Figure 1 (panels B and E), shows that, for both datasets, the first principal component has significantly more structure than a random matrix, whereas the remaining principal components are much less informative and contain less than 20% more information than a purely random matrix.

The analysis described above suggests that both in the CORIELL and CHORI datasets, individuals of European American ancestry lie along a line, and all the variation is concentrated across the first eigenSNP, which corresponds to the first principal component. Although no information about self-reported ancestry was available for the individuals we studied, we can speculate that this axis of variation corresponds to the well-documented axis of northern to southeastern genetic variation in Europe [7], [8], [26], [27], [41], [47]–[50]. Hence we only retained the top principal component for our European American datasets for all further analysis and we interpreted this principal component as the European American ancestry axis. Figure 1 (panels C and F), shows the histogram of the top eigenSNP for individuals in the CORIELL and the CHORI datasets respectively. We would also like to add here a note on the computational efficiency of our methods: our computations are quite efficient and, for example, running PCA on the joint CHORI and CORIELL datasets takes 21 minutes on a standard laptop computer.

**Figure 2:**
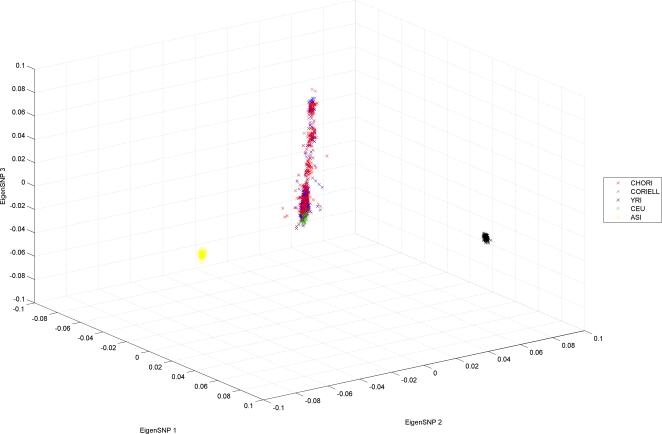
Projection of 958 CHORI, 539 CORIELL, and 270 HapMap individuals on their first, second, and third eigenSNPs. Notice that the individuals of European American ancestry lie along a line with very little deviations toward the Asian and African populations (14 outliers have been removed from this analysis, as described in Methods).

We then compared the structure of the two European American datasets to the structure of the HapMap Yoruba from Ibadan (YRI), CEPH European (CEU), and East Asian populations (CHB and JPT) of the HapMap project. To this end we extracted from the HapMap database genotypes for all SNPs that were also genotyped on our European American samples and computed the top few eigenSNPs of all five populations. Figure 2 shows all 1767 individuals (1497 CHORI and CORIELL plus 270 from HapMap) projected on the first, second, and third eigenSNP of the overall subject-SNP matrix. Adding the HapMap data adds two more axes of variation, one for the African subjects, and one for the Asian subjects. The two large European American samples have similar structure with most individual variation lying across one axis. As expected, they overlap with the CEPH European data. Since outliers were removed as part of a preprocessing step (see Methods), no individuals seem to demonstrate high levels of admixture with non-Europeans. CEPH Europeans form a very tight cluster, which does not seem to encompass the full range of variation observed in European Americans. This also becomes apparent in Figure S2, which focuses on the CHORI, the CORIELL, and the CEPH European datasets only. The fact that the CEPH European samples essentially represent US residents from Utah with Northern European ancestry, corroborates with this picture. Thus, the position of the CEPH European samples in this analysis seems to mark the end of the axis of variation in our European American datasets, which corresponds to Northern European ancestry.

### Using PCAIMs to Capture European American Population Structure

We next tested the feasibility of identifying a small subset of SNPs that could be used to reproduce the structure of the European Americans that we analyzed. Using our algorithm [28] with the number of significant principal components set to one, we selected 100 to 3000 PCAIMs in each dataset in order to predict the ancestry of the European American subjects. As described earlier here, in both European American datasets that we studied, variation lies almost exclusively along the first eigenSNP, which was interpreted as ancestry of the studied individuals.

**Figure 3:**
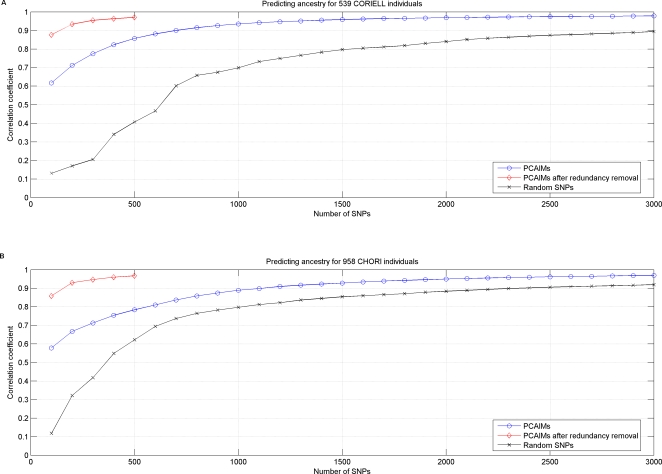
Non-redundant PCAIMs are very good predictors of ancestry. (A) Pearson correlation coefficient between predicted ancestry and “true” ancestry for the 539 subjects in the CORIELL dataset. (B) Pearson correlation coefficient between predicted ancestry and “true” ancestry for the 958 subjects in the CHORI dataset. (For random SNPs, the average over 20 experiments is reported).

In order to evaluate the performance of the PCAIMs that we select, and show that they can be used to preserve the properties of the complete dataset, we computed the first eigenSNP using all available 307,315 SNPs, and compared it to the first eigenSNP using only the selected subset of SNPs. Thus, we essentially predicted the ancestry of each individual by looking at a small subset of SNPs and computing the first eigenSNP of the resulting subject-SNP matrix. Figure 3 shows the Pearson correlation coefficients between “true” and predicted ancestry. In the CHORI dataset, about 1,200 PCAIMs are needed in order to reach a correlation coefficient of above 0.9 and 700 are needed in the CORIELL. Random SNPs perform much worse in the CORIELL and as many as 3,000 random SNPs are needed for the correlation coefficient between true and predicted coordinates of the individuals to reach 0.9. On the other hand, in the CHORI dataset, random SNPs perform worse but overall have comparable performance to PCAIMs (correlation coefficient between “true” and predicted ancestry of individuals is approximately 0.9 with 2,000 SNPs). As we will show in the following section this is due to the redundancy in the markers selected as informative and great savings are indeed possible, after application of our redundancy removal algorithm.

### Removing Redundant PCAIMs

Even though less than 1% (approx. 1,500–2,000) of the total SNPs suffice to predict ancestry in the studied European American datasets with very high accuracy, we still considered this number to be unnecessarily high. This is reinforced by the fact that 2,000–3,000 random SNPs start performing quite well in predicting ancestry. This led us to suspect that the sets of PCAIMs that we were selecting included significant amounts of redundancy. Indeed, we computed all *r* ^2^ values between all pairs of selected PCAIMs for the CHORI dataset, the CORIELL dataset, as well as the joint CHORI-CORIELL dataset. The results are shown in Table 1. Obviously, a large number of pairs are in high LD, and thus a lot of the selected SNPs are redundant.

**Table 1:** Number of pairs of the top 3000 PCAIMs with *r* ^2^ values above 0.1 in the CORIELL dataset, the CHORI dataset, and the joint CORIELL and CHORI dataset.

# pairs with	CORIELL	CHORI	All European Americans
.1≤*r* ^2^<.2	1598	2246	2238
.2≤*r* ^2^<.3	1095	1007	1060
.3≤*r* ^2^<.4	666	708	699
.4≤*r* ^2^<.5	552	612	622
.5≤*r* ^2^<.6	521	457	490
.6≤*r* ^2^<.7	526	496	452
.7≤*r* ^2^<.8	432	304	309
.8≤*r* ^2^<.9	350	194	181
.9≤*r* ^2^<1	589	449	460
exactly 1	115	67	72

There are numerous highly correlated pairs. However, after our redundancy removal step, in the retained 500 PCAIMs there was no pair (in any of the three datasets) with an *r* ^2^ value above 0.2 and only three pairs in the CORIELL dataset with an *r* ^2^ value between 0.1 and 0.2.

**Figure 4:**
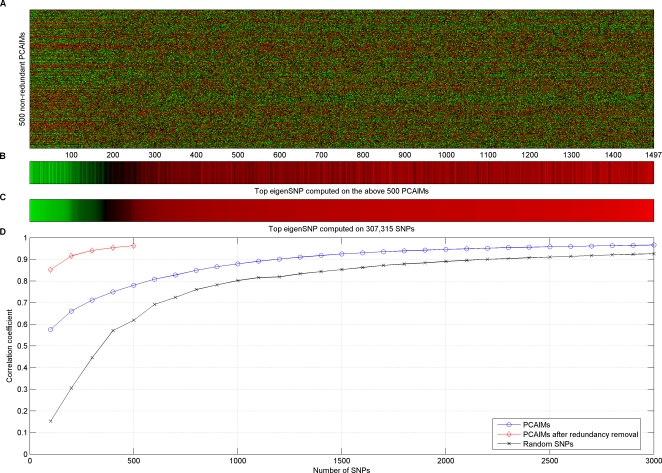
PCA extracts meaningful information from genotype data. (A) Raster plot of the top 500 PCAIMs for all 1497 subjects in the CHORI and CORIELL datasets, after removing redundant SNPs from the top 3000 PCAIMs using the greedy *QR* method. Red and green denotes heterozygotes while homozygotes are black. Individuals are sorted according to their coordinates in the first eigenSNP. (B) The first eigenSNP of the matrix in (A). This vector corresponds to our prediction of ancestry. (C) The first eigenSNP of the matrix of the CHORI and CORIELL subjects on all 307,315 SNPs. This vector is interpreted as “true” ancestry for the individuals. Notice that the two vectors are highly correlated. (D) Pearson correlation coefficient between predicted ancestry and “true” ancestry for the 1497 subjects of European American ancestry using panels of PCAIMs, non-redundant PCAIMs, and random SNPs. Clearly, non-redundant PCAIMs are very good predictors of ancestry.

In an effort to further reduce the number of SNPs that are necessary for ancestry prediction in European Americans and increase genotyping savings, we developed an algorithm that minimizes redundancy from the panels of SNPs that are selected with our scoring algorithm. Applying the redundancy removal procedure described in Methods, we extracted panels of non-redundant SNPs from the top 3,000 PCA-correlated SNPs. We varied the size of these panels from 100 to 500 PCA-correlated non-redundant SNPs. As is shown in Figures 3 and 4, removing redundancy from the selected PCAIMs results in significant savings with as few as 200 SNPs sufficing to accurately predict individual ancestry (with a correlation coefficient above 0.9). Additionally, when we computed all pairwise *r* ^2^ values in the top 500 non-redundant PCA-correlated SNPs for the CORIELL dataset, the CHORI dataset, and the joint dataset, we observed that there was not a single pair of SNPs with an *r* ^2^ value above 0.2 and only three pairs in the CORIELL dataset with an *r* ^2^ value between 0.1 and 0.2. Thus, our algorithm effectively removed redundant SNPs.

In order to generate a potentially more comprehensive list of structure informative SNPs for European Americans, we also analyzed the two datasets jointly (Figure 4 and Figure S3) and tested the efficiency of selected subsets of PCAIMs. Again PCAIMs, after redundancy removal, prove to be quite powerful and as few as 200 can be used to accurately predict the structure of 1497 individuals. Figure S4 shows the scores of selected PCAIMs plotted along each autosome.

### Cross-Validation Experiments

**Figure 5:**
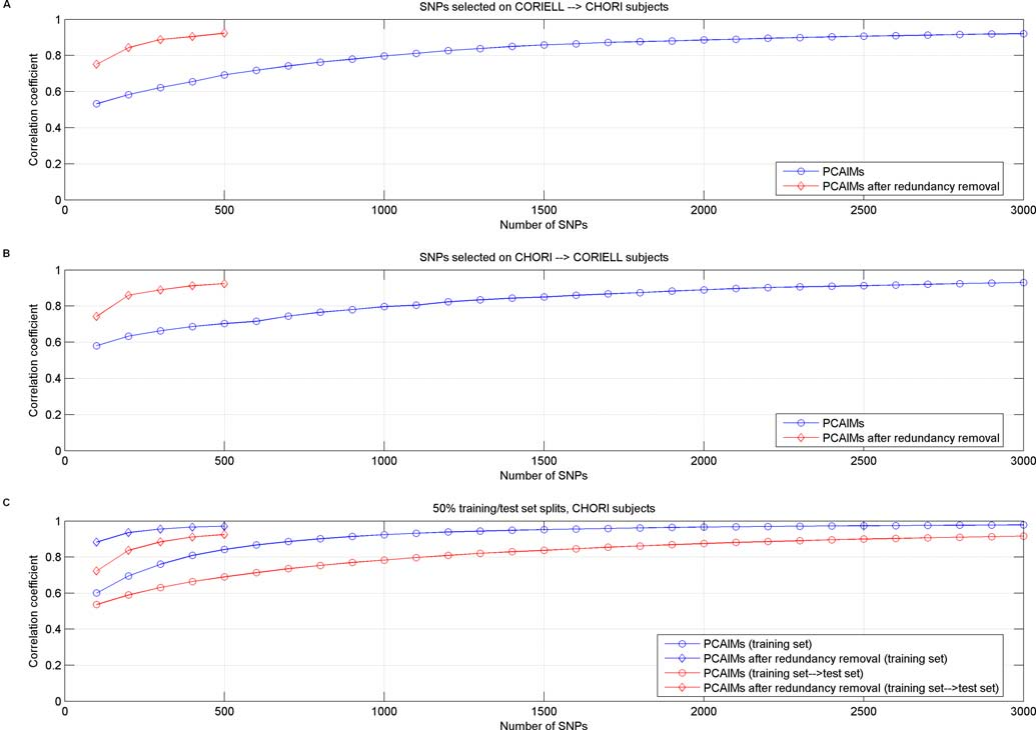
Cross-validation of panels of PCAIMs for ancestry prediction in European Americans. (A) Pearson correlation coefficient between ancestry prediction of CHORI subjects using SNPs selected in the CORIELL dataset, and “true” ancestry of the CHORI subjects. (B) Pearson correlation coefficient between ancestry prediction of CORIELL subjects using SNPs selected in the CHORI dataset, and “true” ancestry of the CORIELL subjects. (C) Split of the CHORI dataset in 50% training and 50% test set. Pearson correlation coefficient between ancestry prediction of test set subjects using SNPs selected in the training set, and “true” ancestry of the test set subjects. Results are reported over 20 splits.

In order to further evaluate our results, we split the CHORI dataset in 50% training set and 50% test set, selected PCAIMs in the training set (with and without redundancy removal) and used these SNPs to predict the ancestry of the individuals in the test set (Figure 5). The PCAIMs selected in the training set achieve comparable performance in the test set. We repeated the same experiment in the Coriell dataset, as well as with different split sizes for both datasets (e.g., 80% training, 20% testing) and obtained similar results (data not shown).

We then cross-validated our results by using the PCAIMs selected in one European American dataset for prediction of structure in the other European American dataset (Figure 5). We found that as few as 500 of the top PCAIMs selected in each dataset suffice for the accurate prediction of structure in the other dataset. The actual overlap between the top 500 PCAIMs selected in each sample is relatively small (6.8% or 34 SNPs). Of course this is still highly significant compared to the overlap between two random sets of 500 SNPs selected from approximately 307,315 SNPs, which is 0.16% with a standard deviation of 0.07%. Additionally, some amount of linkage disequilibrium can be observed between the top 500 PCAIMs selected in each of the two datasets. We computed *r* ^2^ values for all possible pairs and found 44 pairs of SNPs that had *r* ^2^ of at least 0.1, with an average value of 0.43. These pairs are in addition to the 34 pairs of overlapping SNPs between the two sets. So, it seems that there exist different sets of SNPs that are mildly correlated and yet provide similar information about the structure of the European American population.

### Correcting for Stratification using PCAIMs

Finally, we examined the extent to which small subsets of PCAIMs can be used for correction of stratification in the setting of an association study. Following the model and parameters used by Price et al. [19], we first simulated an admixed population with 1000 members genotyped on 100,000 SNPs, originating from two ancestral populations that are relatively closely related. In particular, the average *F_st_* between SNPs in the ancestral populations was set to 10^−2^ (see Methods and Text S1 for details). This gave us the advantage of knowing the “true” ancestry of each simulated individual, while at the same time constructing a simulated population whose structure is quite similar to the structure of our European American datasets. By looking at the singular values associated with the top eigenSNPs of the subject-SNP matrix, as well as by applying our permutation test, one principal component was deemed significant. Thus, in this simulated dataset, again individual variation lies across the first eigenSNP (Figure S5). In fact, if this eigenSNP is used as a predictor for ancestry, the Pearson correlation coefficient between true and predicted ancestry coefficient over all individuals is 0.9967. As expected, PCAIMs work extremely well for the prediction of ancestry in the simulated data and as few as 100 to 400 PCAIMs are enough to accurately predict the ancestry of each individual (Table 2). In fact the Pearson correlation coefficient between true (recall that in this case we know the actual ancestry) and predicted ancestry, calculated by looking at the first eigenSNP of a matrix containing 100, 200, and 400 PCAIMs is 0.9102, 0.9478, and 0.9690 respectively. Using this particular method of constructing simulated SNPs results in mostly uncorrelated SNPs. Consequently, in this synthetic dataset, our redundancy removal algorithm did not improve our results.

**Table 2:** Using PCAIMs for stratification correction in conjuction with EIGENSTRAT's algorithm.

**Admixed** (*r* = 2)	No correction	400 PCAIMs	200 PCAIMs	100 PCAIMs
*Random*	0.0002	0.0001	0.0001	0.0001
*Stratified*	0.1246	0.0001	0.0001	0.0002
*Causal*	0.5203	0.4735	0.4716	0.4790
**Admixed** (*r* = 3)	No correction	400 PCAIMs	200 PCAIMs	100 PCAIMs
*Random*	0.0005	0.0001	0.0001	0.0001
*Stratified*	0.6182	0.0001	0.0001	0.0003
*Causal*	0.5110	0.4141	0.4189	0.4340

The first column shows the proportion of random, stratified, and causal SNPs that are identified as causal using the Armitage's trend test with a cut-off *p*-value of 10^−4^. The remaining columns show the respective proportions after stratification correction using 100, 200, and 400 PCAIMs.

In order to test if small subsets of PCAIMs could be used for correction for stratification, we simulated association studies with sets of 100,000 random, extremely stratified, and truly causal SNPs (see Methods for details) for 10 different datasets. We first replicated the results of Price et al. [19] in order to correct for stratification using the top 10 principal components computed on all 100,000 SNPs without significant loss in power (Table 2). We then selected subsets of 100 to 400 PCAIMs in order to predict the ancestry of all 1,000 individuals. We proceeded to correct for stratification by removing (projecting out) our ancestry prediction from each SNP and then ran the Armitage trend test to the resulting SNPs. (This is essentially the algorithm implemented in EIGENSTRAT.) We measured the percentage of correlations found using the Armitage-trend test in each scenario and report the results before and after stratification correction in Table 2. According to our findings, as few as 100 PCAIMs (instead of 100,000 SNPs) efficiently remove false correlations with disease, while largely maintaining the power of the study.

## Discussion

We have identified small sets of structure informative markers for the European American population through the direct investigation of European American samples and without depending on any assumptions about the ancestry or admixture proportions of the studied individuals. We have analyzed two independent datasets of European Americans, representing a total of almost 1500 individuals genotyped for more than 300,000 SNPs spanning the entire autosomal genome, and we have demonstrated that as few as 200 SNPs (PCAIMs), carefully selected with our methodology, can be used to very accurately predict the genetic structure of European Americans as identified by PCA. The cross-validation experiments that we have performed verify the validity of our approach. Investigating the European American population directly for the selection of structure informative genetic markers results in SNP panels that provide a direct reflection of the complex patterns of sub-structure and admixture in European Americans.

The analysis of the admixed European American population for the selection of structure informative markers was made possible through the application of the unsupervised method that we have recently introduced for the selection of PCA-correlated SNPs or PCAIMs [28]. As we have previously described, PCAIMs selection can be carried out without any need for prior knowledge of individual ancestry, and is thus feasible in admixed populations without having to trace the origin of the studied individuals or hypothesize about admixture proportions [28]. This is not possible when using allele-frequency based methods for the selection of AIMs like *δ*, *F_st_* or informativeness for assignment [20]–[25].

An additional important contribution of the present study is the novel algorithm that we developed for the removal of redundancy from a given set of structure informative markers. All existing algorithms for AIM selection (e.g., *δ*, *F_st_*, informativeness, as well as PCAIMs), could potentially suffer from selecting a large number of redundant SNPs. For example, consider the simple scenario where a SNP is assigned a high score, and many SNPs are in very high LD with this SNP. Then, they will also be assigned very high scores, and thus will be chosen as AIMs, even though they are clearly redundant. Thus, if the task at hand is to select a minimal set of AIMs (as is the case in our work), a second step is necessary in order to remove redundant AIMs. Given the large number of SNPs (many of which are in LD) in genome-wide scans over the last year, this is certainly a significant concern. Notice for example the fact that, in the datasets we studied, fewer than 10,000 such pairs exist (Table 1), and even though this is a proportionally small percentage out of the 
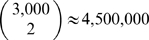
 possible pairs it still significantly increases the number of PCAIMs needed to perfectly capture the structure of the data.

In order to address this deficiency, we propose an efficient and accurate algorithm that filters out redundant SNPs from the set of PCA-correlated SNPs. The proposed algorithm emerges by reducing the redundancy removal problem to a well-known problem in the numerical linear algebra community, the so-called Column Subset Selection Problem, as defined earlier here. As we have shown here, applying this algorithm significantly increases genotyping savings, reducing the number of SNPs needed for structure identification almost by six-fold. This method for redundancy removal can be applied to any set of SNPs in order to select a minimally correlated subset. We should note that the proposed algorithm does not necessarily return the absolutely optimal solution to the Column Subset Selection problem. Formal mathematical bounds regarding the accuracy of the algorithm do exist, arguing that the selected subset of columns (i.e. SNPs) provides an almost optimal solution [46]. Further discussion on this is perhaps beyond the scope of this paper. Alternatives that take into account LD estimation and physical distance could also be considered. Notice however, that our method is parameter free and achieves effective redundancy removal in a single step.

The two independent samples of European Americans that we studied show comparable structure, while the CEPH European Americans represent only a small fraction of the entire breadth of variation that we encountered in these large datasets. We are able to faithfully reproduce this fine structure using as few as 200 PCAIMs. We found that the SNPs selected in the first European American dataset we studied could be successfully applied in the second dataset and vice versa; however, the absolute actual overlap was relatively small (although significantly higher than what expected by chance alone) suggesting the possibility that many different such subsets of informative SNPs exist.

Several other studies have explored intra-European and European American genetic variation. Classic gene frequency [41],[47], Y-chromosome [48] or mitochondrial variation [49],[50] as well as whole-genome studies [7],[8] generally agree on a coarse separation of European populations along a northern to southeastern axis. Seldin et al. [7] analyzed 5705 SNPs from the ILLUMINA Linkage IV panel to calculate informativeness for assignment, and identified 400 SNPs that could be used in order to broadly cluster the populations they studied to northern and southern Europeans. Bauchet et al. [8] studied 10,000 SNPs (Affymetrix 10K panel) and about 100 individuals from 12 European populations and concluded that at least 1,200 high Fst SNPs were needed in order to achieve a similar clustering of northern versus southern Europeans. Our results build on these papers, using large datasets of genomewide markers, and an algorithm that can explicitly identify informative markers from admixed populations without knowledge of the ancestral populations. Finally, we demonstrate that our markers are valid across large European American studies. We found almost no overlap between the markers that we identify as ancestry informative and those reported in the above mentioned studies of European populations [7],[8] (data not shown). This was to be expected since all three studies analyze different datasets and different populations. Notice, that even between these two previous studies, there is very little overlap between the panels of SNPs reported as ancestry informative.

Very recently, two studies parallel to ours, used several genomewide sets of markers in European Americans to derive small subsets of European American AIMs [26],[27] (see also Tables S1 and S2). An important difference between these studies and ours is the fact that we employed a previously validated algorithm for the selection of AIMs [28], that operates directly on raw data without the need for intermediate steps (i.e., artificial assignment of individuals to clusters, depending on candidate genes for local natural selection, etc.). As we have seen here, and as others have also discussed [26],[51], individual variation in the European American population seems to lie along a continuum rather than in distinct clusters. Thus, the method we have used here would be easier to generalize to diverse datasets without access to ancestral populations. Another important difference of our study, is the fact that, as we have also discussed previously here, we have employed a novel, linear algebra based algorithm in order to select the least correlated SNPs as part of our structure informative panel thus increasing the efficiency of our informative SNP sets. In comparison, Price et al. [26] and Tian et al. [27] reduced redundancy by applying measures based on physical distance.

Our results are consistent with the findings of Price et al. [26] and Tian et al. [27], who also demonstrated that the vast amount of inter-individual variation in European Americans lies across a single axis. In concordance with what we have also described here, Tian et al. [27] mention that the first principal component in their study accounted for greater than five-fold the variance of the second principal component (percentage of total variance according to their analysis is 42.42% for the first principal component, and 8.32% and 6.66% for the second and third respectively [27]). Both [26],[27] analyzed individuals of known ancestry and they could distinguish a cluster comprising of individuals of known Ashkenazi Jewish origin. Price et al. [26] argue that an additional principal component is needed in order to discern this line of ancestry. However, both of these studies included large subpopulations with known Ashkenazi Jewish ancestry. For example in Price et al. [26], in the inflammatory bowel disease (IBD) study, 43% of included individuals self-reported as Ashkenazi Jewish (78% among individuals of known ancestry in this sample). In Tian et al. [27] 28% of the population analyzed was of known Ashkenazi heritage (for comparison, 2% of the general US population self-reports as Ashkenazi Jewish [26],[52],[53]). Thus, the larger Ashkenazi Jewish population in the Price et al. [26] study likely helped to bring out an additional principal component for this population.

It is likely that there were Ashkenazi individuals in the datasets that we studied; however, they probably constituted a smaller fraction of the overall population. Analyzing the top two eigenSNPs, corresponding to the top two principal components in our datasets (data not shown), a small cluster of individuals becomes visually apparent. (A similar figure is shown in [26] for the PD dataset which corresponds to our CORIELL dataset.) As we have no information on individual ancestry, we cannot infer the origin of the individuals in this small cluster. Interestingly, this very small cluster (which might correspond to Ashkenazi individuals in our population), is already reasonably separated from the remaining European Americans along the top eigenSNP, at least in the datasets that we studied. This observation is consistent with Tian et al.'s report [27]; in the sample they studied, the mean score of the top eigenSNP for individuals of known Ashkenazi Jewish ancestry, lay at one end of the distribution (0.045 for Ashkenazi Jewish individuals, followed by 0.022 for Greeks and 0.015 for Italians). This explains why our permutation test only detects the first principal component as statistically significant: our test removes from the data the amount of information that has already been captured by principal components that were deemed significant.

The SNP panels proposed by Price et al. [26] and Tian et al. [27] perform very well when tested on our samples of European Americans (Table S1). The Tian et al. [27] panel of ancestry informative SNPs was selected by calculating *I_n_* [25] for two discrete clusters; Ashkenazi Jewish individuals (as representatives of southeastern or rather mediterranean European ancestry) and northern Europeans. The SNPs selected by this method perform exceptionally well (comparably to our SNP panel) to recreate the individual ancestry in our analysis (see Table S1). This suggests the fact that most of the variation between southeastern and northern European ancestry is captured by the difference between Ashkenazi versus northwestern European ancestry. On the other hand, we could not fully test the SNPs proposed in the second study [26], since they had not all been genotyped in our datasets. Price et al. [26] proposed 300 SNPs as informative for European American ancestry (100 discerning the northern European versus southeastern cluster and 200 differentiating the southeatern versus Ashkenazi Jewish clusters). Out of these SNPs, 141 had also been genotyped in the datasets we studied. However, using these 141 SNPs [26], results in a correlation coefficient of 0.75 between true and predicted individual variation in our combined CORIELL and CHORI datasets (Table S1).

There is generally little (although far greater than chance) overlap between the lists of structure informative SNPs identified by each of these three studies (see Table S2). The greatest overlap is found between the panel we propose here and the 1,441 SNPs proposed by Tian et al. [27] as distinguishing between northern European and Ashkenazi Jewish ancestry; out of the 1,419 SNPs that were also included in our analysis, 36 were among the 500 top informative SNPs that we selected in the analysis of our combined European American datasets. The overlap between the informative SNPs proposed by Price et al. [26] and the other two studies is even smaller, partly due to the fact that we could only test 141 out of the 300 proposed SNPs (see Table S2). In any case, as we have also suggested earlier here, it is probably not surprising that there exist more than one subsets of SNPs describing European American population structure.

It is now clear that European derived populations are not homogeneous and recent studies have emphasized the problem of population stratification in genetic association studies which may lead to false positive associations with disease or mask true correlations [5],[19]. As association studies of thousands of individuals are starting to become increasingly common [9]–[14], population stratification will undoubtedly pose a serious challenge. Various methods have been proposed to tackle the problem [16], [18], [19], [54]–[60]. Among them, PCA-based stratification correction tools seem particularly attractive, since they are computationally efficient and are not overly conservative. Moreover, such methods do not demand the use of discrete clusters, which as we have discussed earlier here may be an over-simplification, especially in the case of admixed populations.

We have replicated the analysis of simulated data in [19] and experimentally demonstrated how our method can complement PCA-based stratification correction methods. Using as few as 100 to 200 PCAIMs, we achieved almost perfect stratification correction with virtually no loss in power. In comparison previous simulation studies [19] have shown that as many as 5,000 randomly selected SNPs would be needed to reach similar performance, while 20,000 random SNPs were needed in a real dataset [19]. Comparing the accuracy of ancestry prediction in the simulated and real data we have studied we can extrapolate that as few as 200 SNPs could be enough for stratification correction in real data (reaching a Pearson correlation coefficient above 0.9 between “true” and predicted ancestry across the second eigenvector). While the selection of AIMs for stratification correction may be unnecessary for teams of investigators that undertake an initial genome-wide association study and can afford genotyping of very dense maps of markers, the use of AIMs for stratification correction becomes of critical importance in two-stage study designs, (where replication of initial findings is sought in large independent samples), or studies following the candidate gene approach. In such cases, our methods can greatly facilitate association studies in admixed populations, reducing significantly the genotyping costs needed to ensure correction for stratification.

We would like to point out that, the sets of European American AIMs that we and others [7],[8],[26],[27] have identified, are representative of the full genetic structure in the European American population, only to the extent that the samples analyzed in each of these studies are deemed truly representative of the entire European American population. It will be important to study European American population structure with even larger datasets of carefully sampled individuals. Interestingly, in Tian et al. [27], the effect of stratification on the case-control study of rheumatoid arthritis was mostly due to a difference in Irish ancestry. This suggests that different European American studies will have to exercise caution in detecting and adjusting for ancestry, since the components/axes that affect ancestry are likely to vary from study to study depending on the phenotype and the region sampled.

In summary, we are proposing a small set of SNPs that can successfully capture the structure of the European American population samples we studied, as identified by PCA. We identified this minimal set of structure informative SNPs (PCAIMs) by applying a novel redundancy removal algorithm that will undoubtedly increase genotyping savings in many different research scenarios. Lists of the sets of markers that we have identified as well as an implementation of our algorithms are available online at http://www.cs.rpi.edu/drinep/EUROAIMs/. These panels of SNPs will serve as useful tools in the discovery of susceptibility genes for common complex disorders and can spark interesting questions in population genetics regarding the possible role of natural selection in the regions of the genome harboring these polymorphic sites.

## Supporting Information

Figure S1 Plot of 970 CHORI, 541 CORIELL, and 270 HapMap subjects on their first, second, and third eigenSNPs. Five CHORI pairs (ten CHORI subjects) were suspiciously similar and were excluded prior to this plot. The red squares represent the 14 individuals (12 CHORI and two CORIELL) that were excluded from further analysis. Notice that these individuals tend to have atypical degrees of Asian and African ancestry.
(0.09 MB PDF)

Figure S2 Plot of 960 CHORI, 539 CORIELL, and 90 CEPH European HapMap subjects on their first eigenSNP. Notice CEPH Europeans form a tight cluster that does not seem to encompass the full variation of European American populations.
(0.04 MB PDF)

Figure S3 Using non-redundant PCAIMs to predict the first eigenSNP in European American datasets. The first eigenSNP of 1497 European Americans (CHORI and CORIELL datasets) analyzing 307,315 SNPs, plotted against the predicted first eigenSNP of each individual with 200 and 300 non-redundant PCAIMs.
(0.18 MB PDF)

Figure S4 PCA scores of 307,315 studied SNPs in the combined CHORI and CORIELL datasets plotted along each autosome. The blue “x” marks the top 3,000 PCAIMs, while the red squares denote the top 500 PCAIMs after redundancy removal. Notice the different scale of the Y axis for each chromosome. (A) Chromosomes 1–5, (B) Chromosomes 6–10, (C) Chromosomes 11–15, (D) Chromosomes 16–20, (E) Chromosomes 21 and 22.
(0.68 MB PDF)

Figure S5 A simulated admixed population of 1000 subjects genotyped on 100,000 SNPs. The admixed population emerges from two ancestral populations with an average F_st_ of 10^−2^, as described in Methods.
(0.03 MB PDF)

Table S1 Performance of published European American AIMs, for population structure prediction in the datasets we studied.
(0.03 MB DOC)

Table S2 Overlap between European American AIMs proposed in studies of European American datasets.
(0.03 MB PDF)

Text S1 Supplementary note.
(0.06 MB PDF)
