# Supplementary figures and images for: Tracing Sub-Structure in the European American Population with PCA-Informative Markers
Source: PLoS Genet. 2008 Jul 4;4(7):e1000114. doi: 10.1371/journal.pgen.1000114 (PMC2537989; doi:10.1371/journal.pgen.1000114)

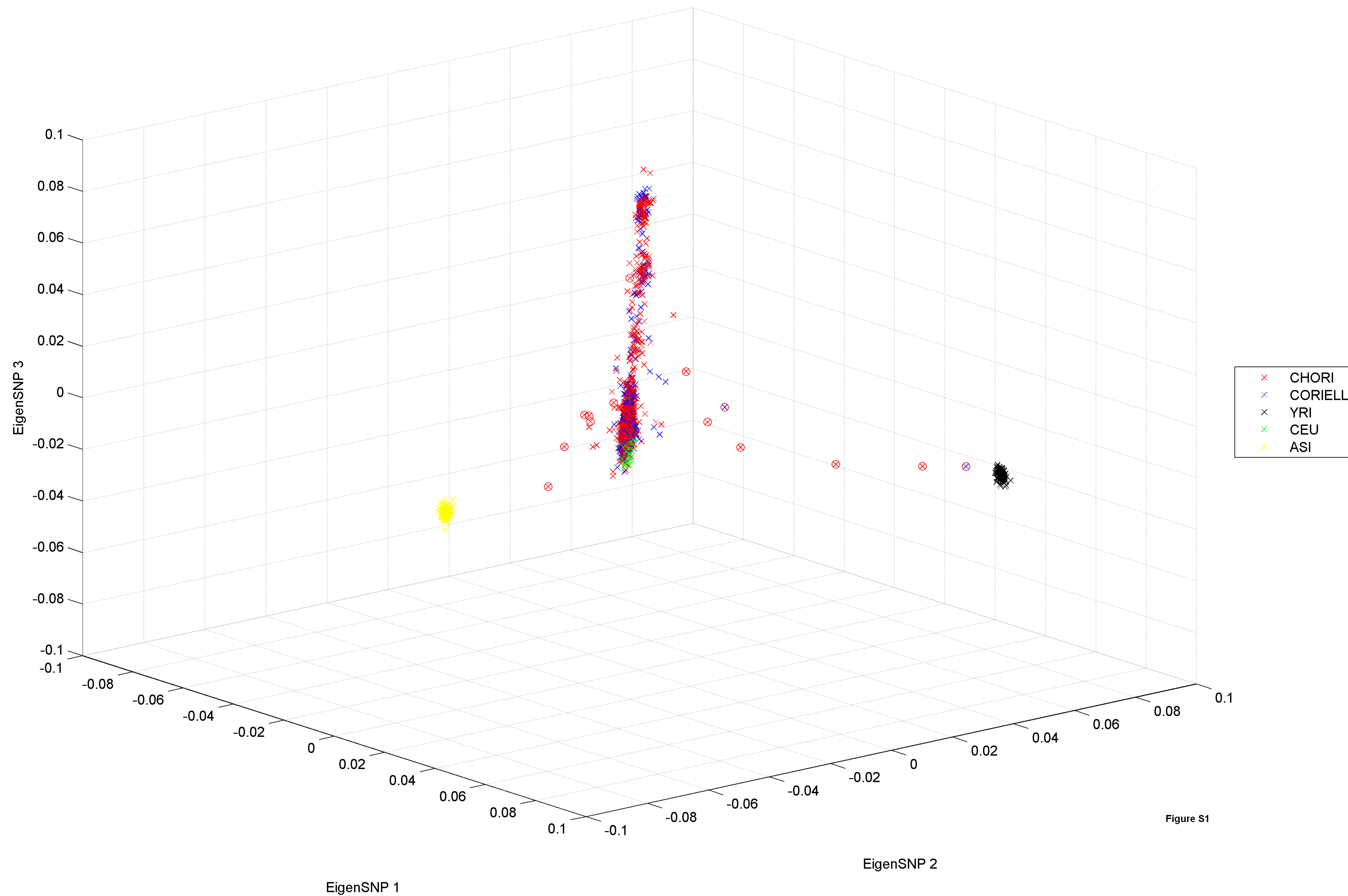

Supplement: Figure S1 — Plot of 970 CHORI, 541 CORIELL, and 270 HapMap subjects on their first, second, and third eigenSNPs. Five CHORI pairs (ten CHORI subjects) were suspiciously similar and were excluded prior to this plot. The red squares represent the 14 individuals (12 CHORI and two CORIELL) that were excluded from further analysis. Notice that these individuals tend to have atypical degrees of Asian and African ancestry. (0.09 MB PDF) [file pgen.1000114.s001.pdf]

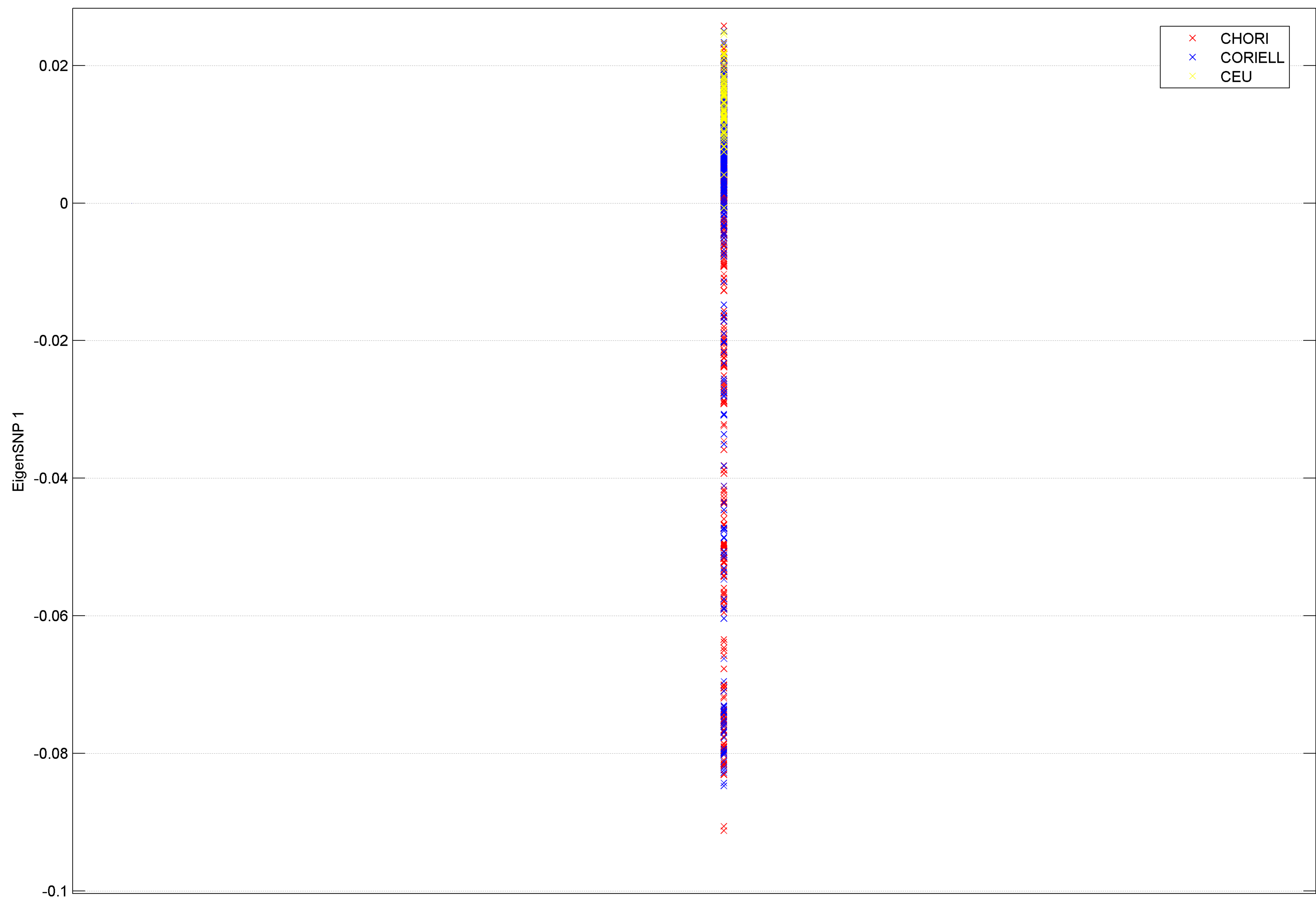

Figure S2

Supplement: Figure S2 — Plot of 960 CHORI, 539 CORIELL, and 90 CEPH European HapMap subjects on their first eigenSNP. Notice CEPH Europeans form a tight cluster that does not seem to encompass the full variation of European American populations. (0.04 MB PDF) [file pgen.1000114.s002.pdf]

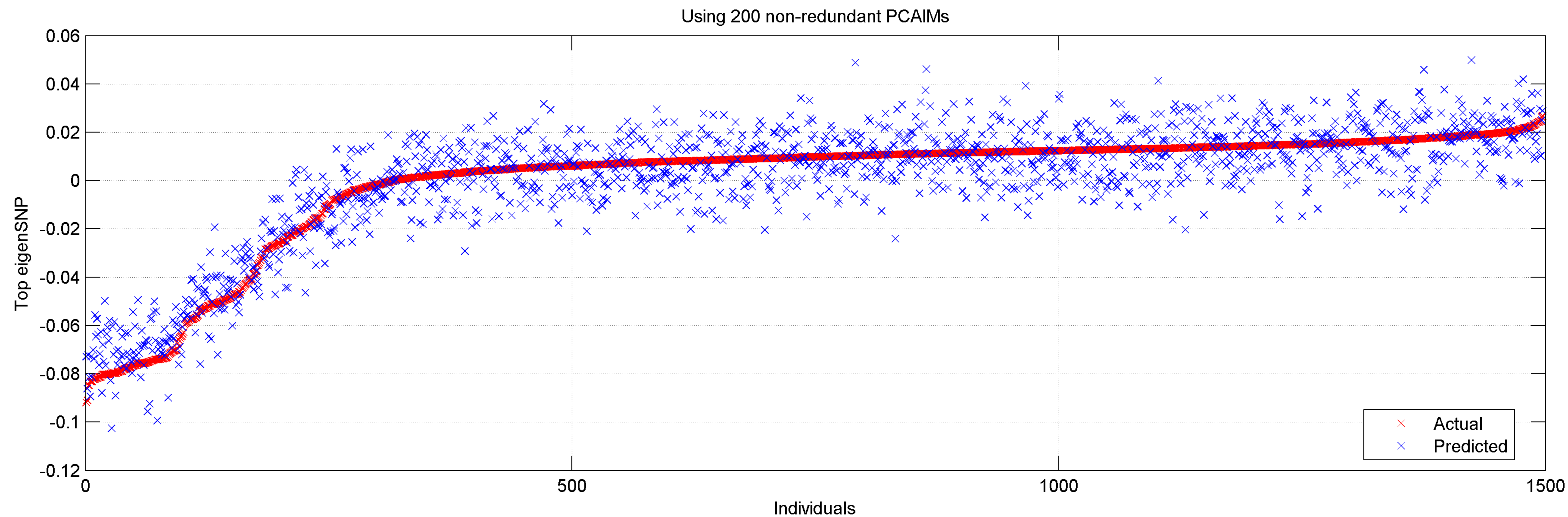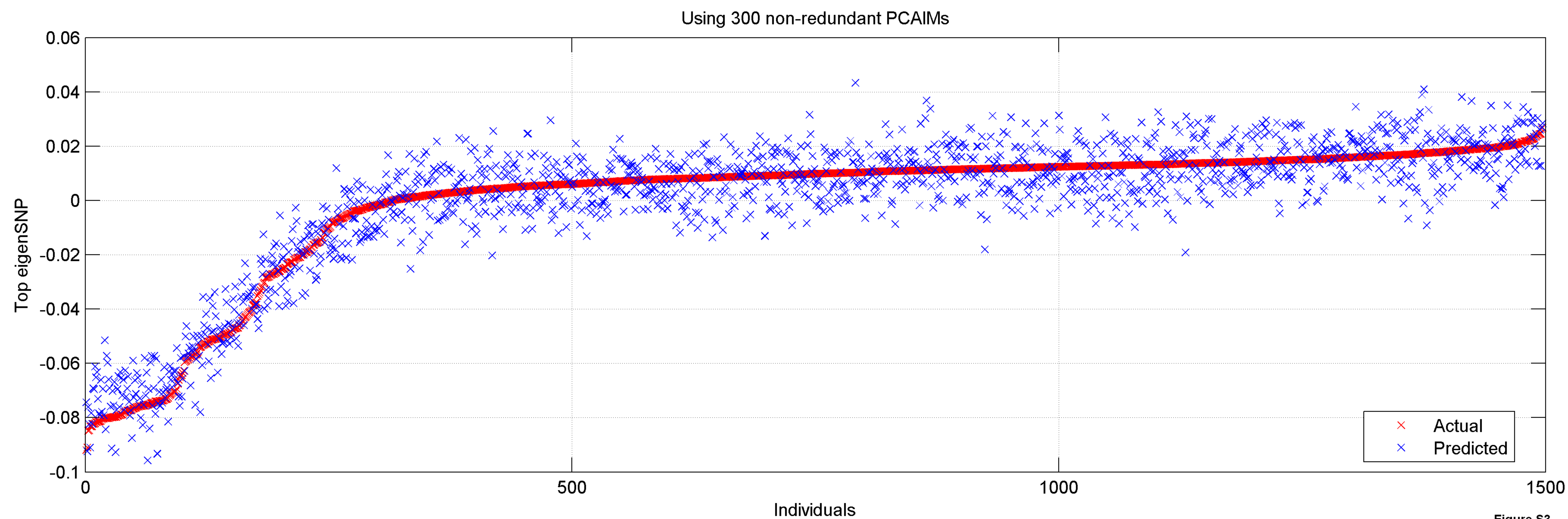

Figure S3

Supplement: Figure S3 — Using non-redundant PCAIMs to predict the first eigenSNP in European American datasets. The first eigenSNP of 1497 European Americans (CHORI and CORIELL datasets) analyzing 307,315 SNPs, plotted against the predicted first eigenSNP of each individual with 200 and 300 non-redundant PCAIMs. (0.18 MB PDF) [file pgen.1000114.s003.pdf]

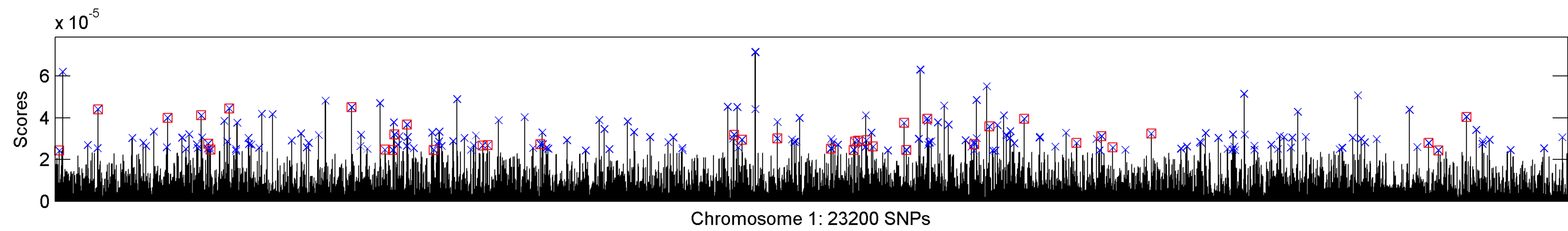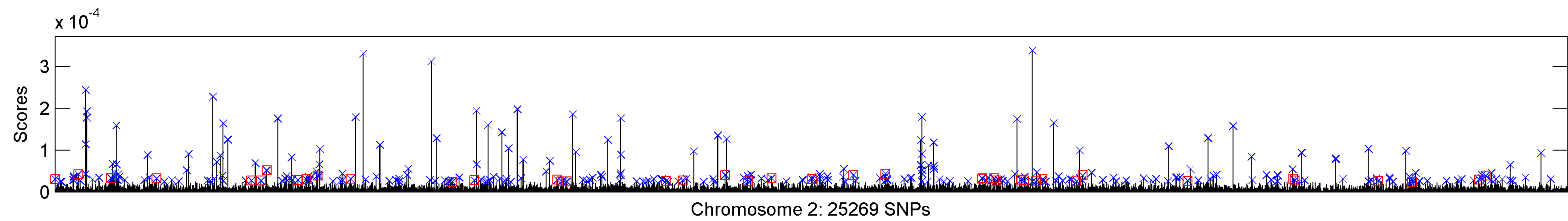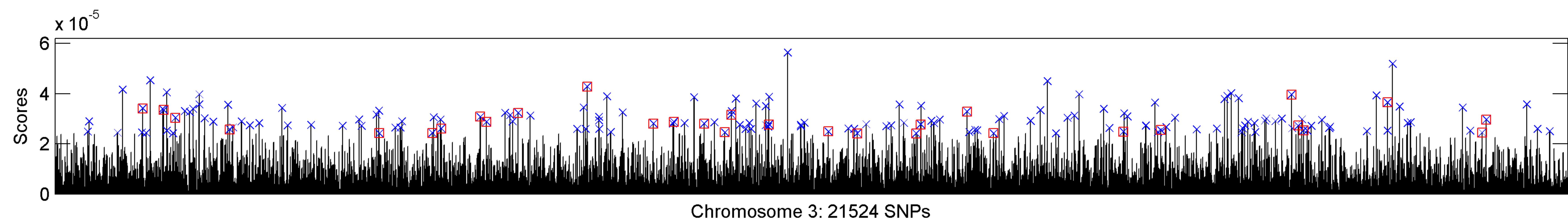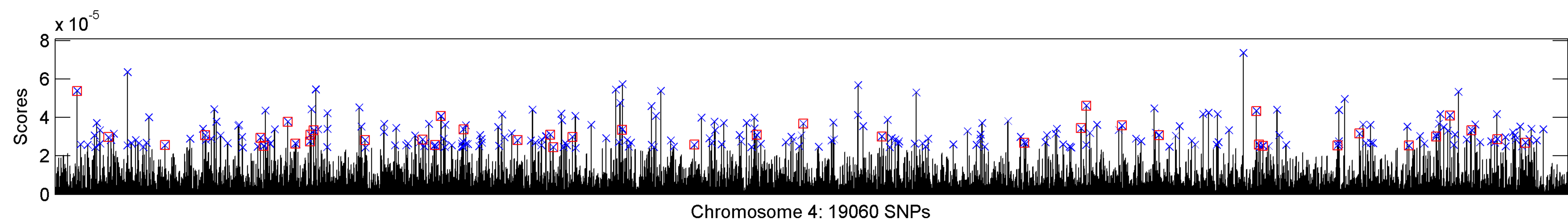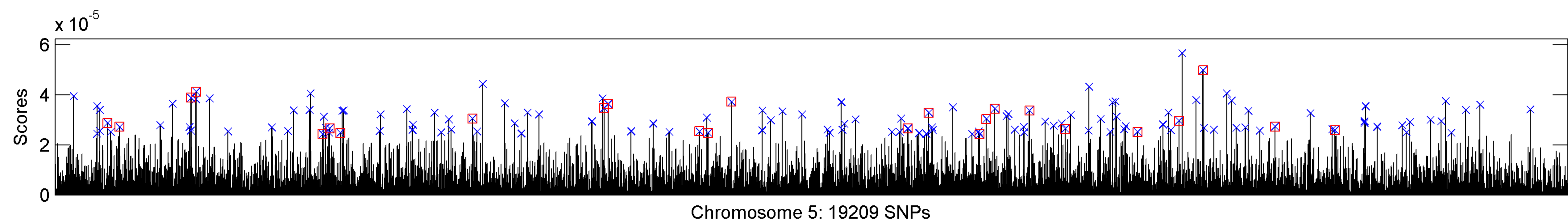

Figure S4A

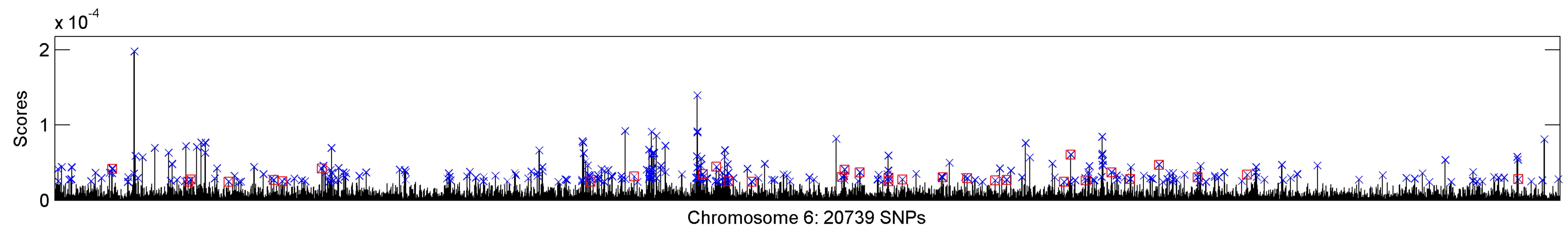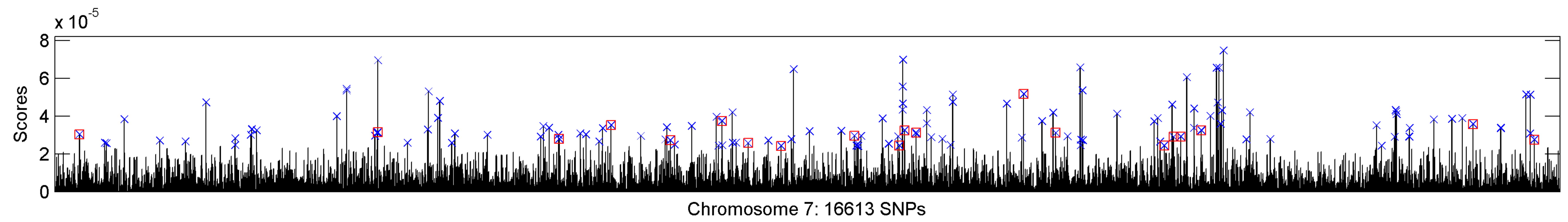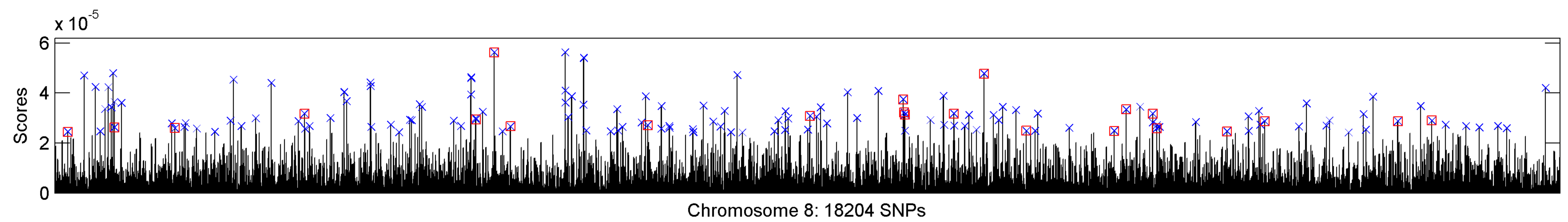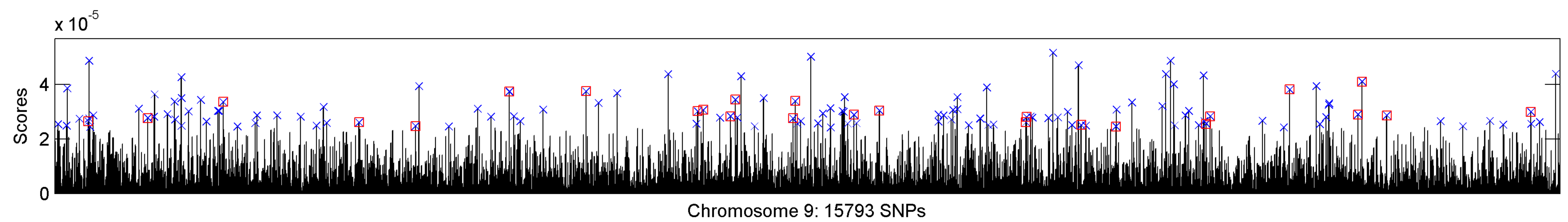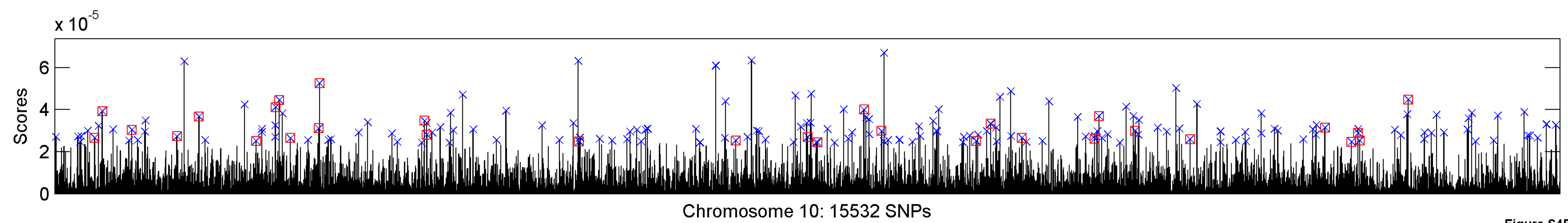

Figure S4B

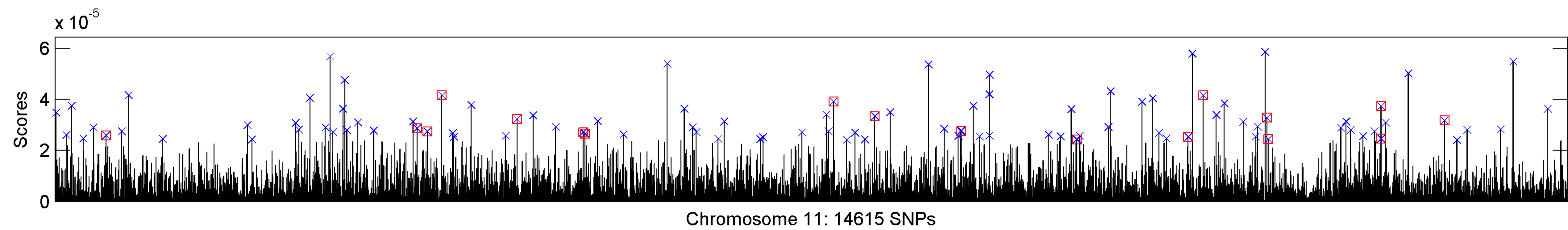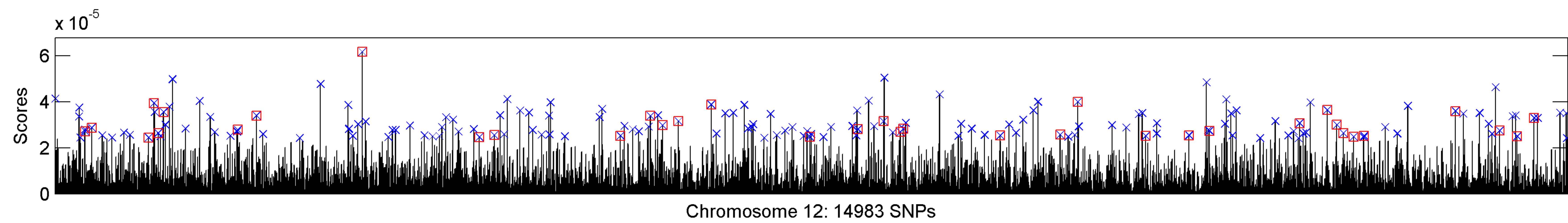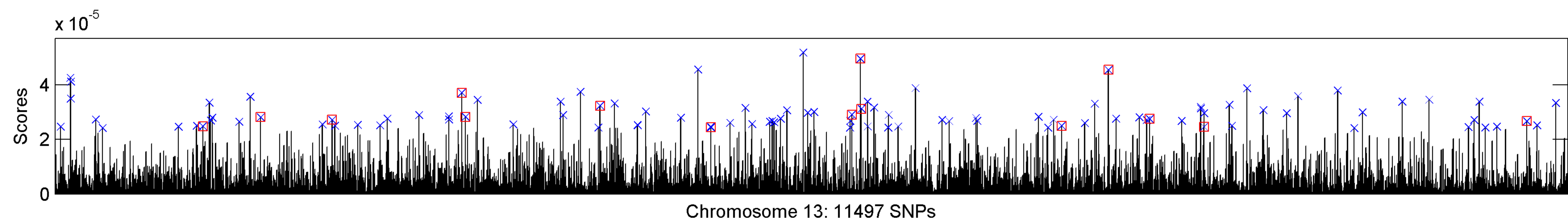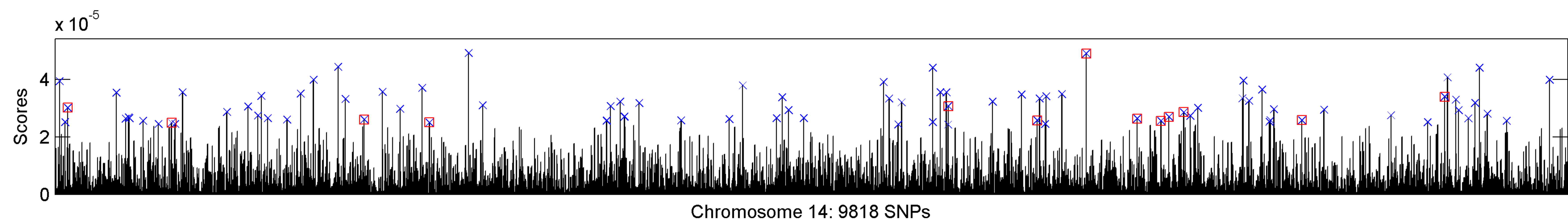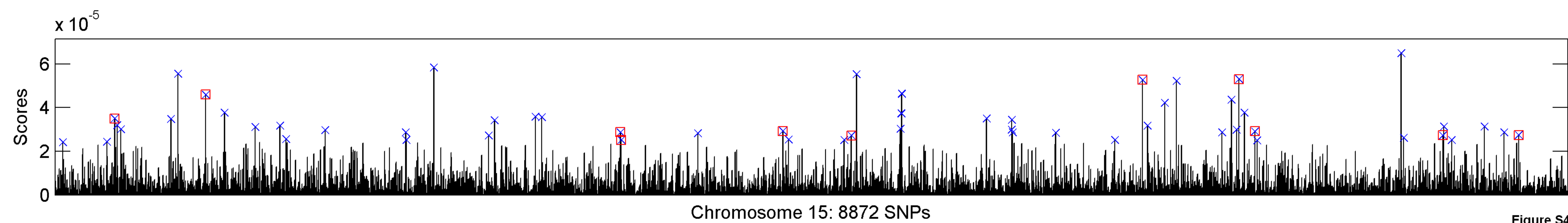

Figure S4C

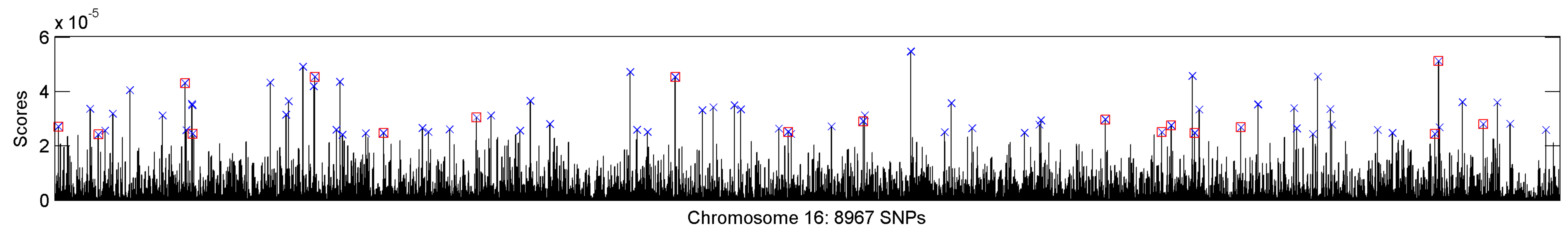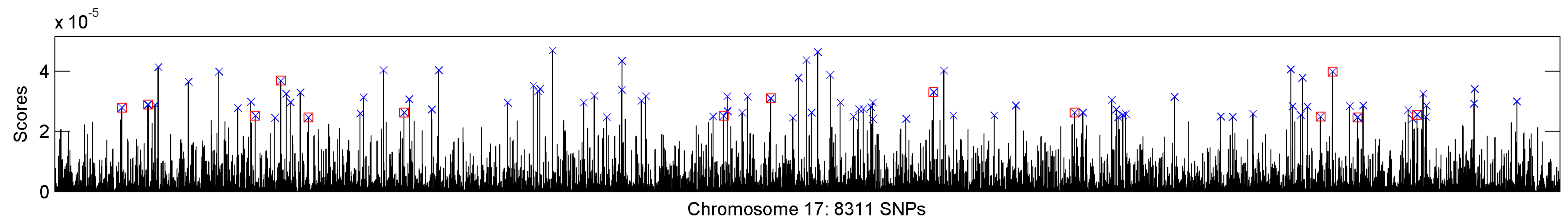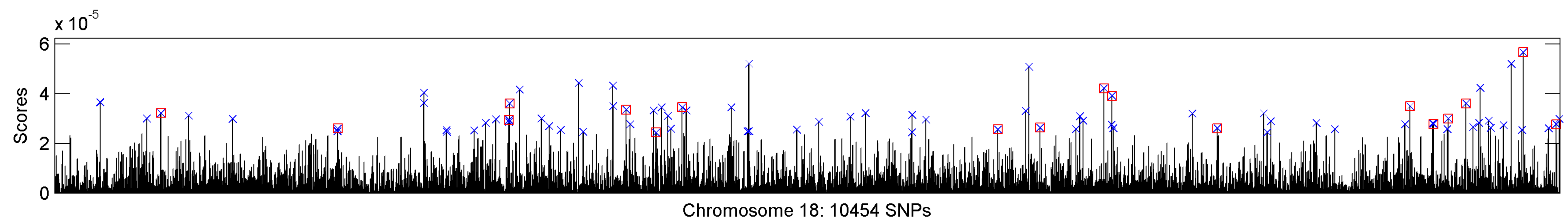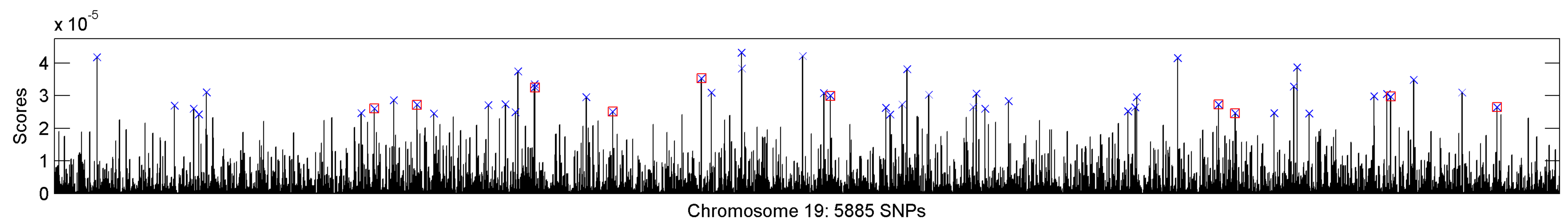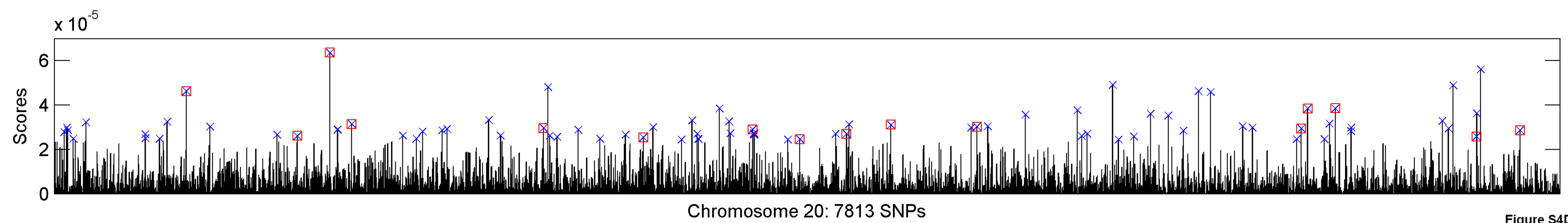

Figure S4D

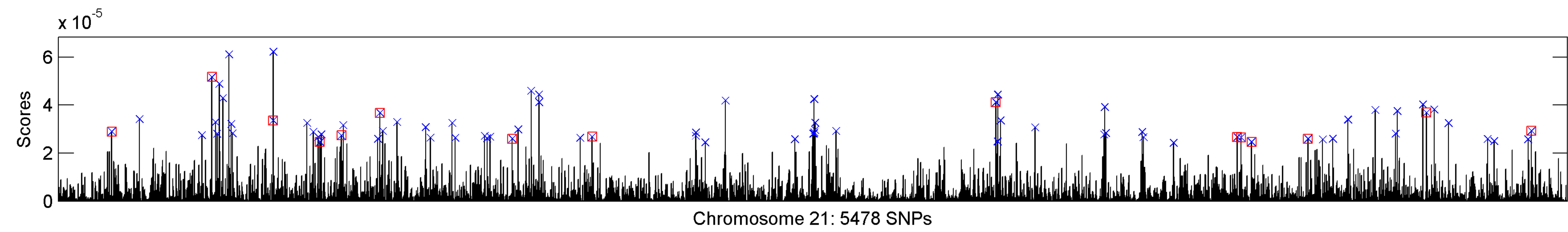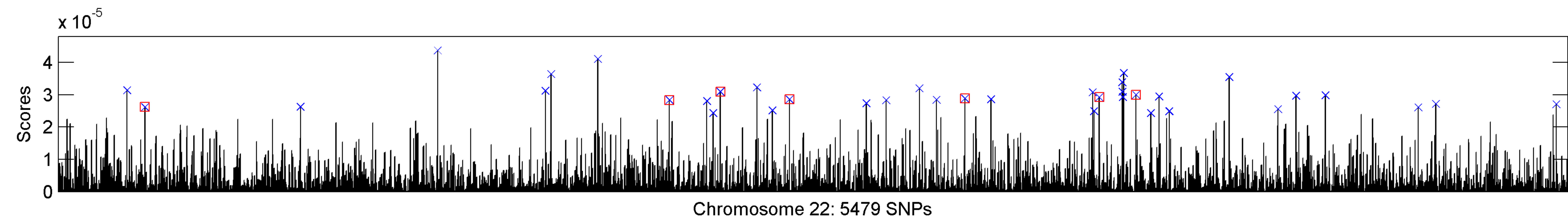

Figure S4E

Supplement: Figure S4 — PCA scores of 307,315 studied SNPs in the combined CHORI and CORIELL datasets plotted along each autosome. The blue “x” marks the top 3,000 PCAIMs, while the red squares denote the top 500 PCAIMs after redundancy removal. Notice the different scale of the Y axis for each chromosome. (A) Chromosomes 1–5, (B) Chromosomes 6–10, (C) Chromosomes 11–15, (D) Chromosomes 16–20, (E) Chromosomes 21 and 22. (0.68 MB PDF) [file pgen.1000114.s004.pdf]

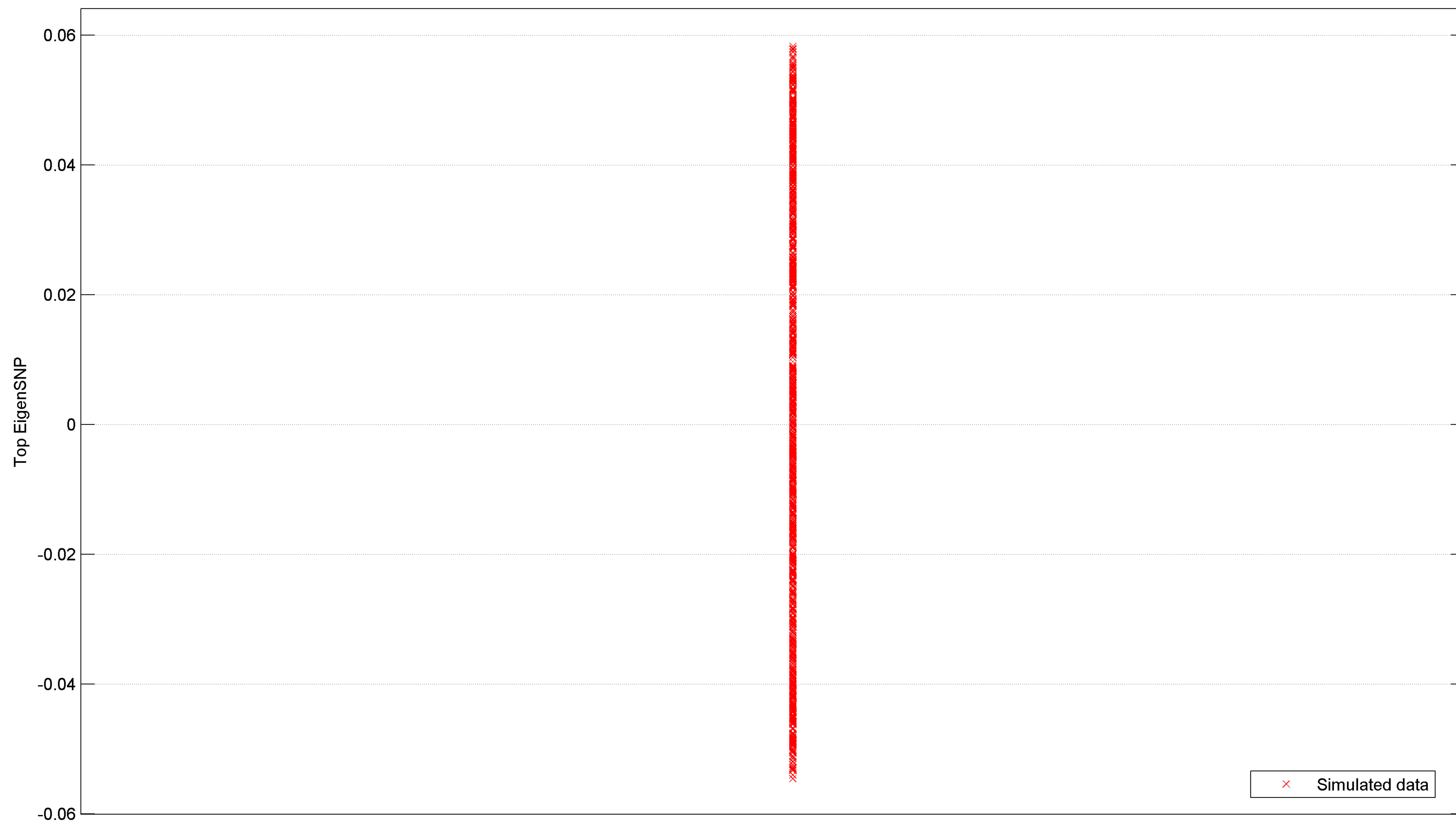

Figure S5

Supplement: Figure S5 — A simulated admixed population of 1000 subjects genotyped on 100,000 SNPs. The admixed population emerges from two ancestral populations with an average Fst of 10−2, as described in Methods. (0.03 MB PDF) [file pgen.1000114.s005.pdf]
