# Supplementary material for: Tracing Sub-Structure in the European American Population with PCA-Informative Markers
Source: PLoS Genet. 2008 Jul 4;4(7):e1000114. doi: 10.1371/journal.pgen.1000114 (PMC2537989; doi:10.1371/journal.pgen.1000114)
Supplement: Table S1 — Performance of published European American AIMs, for population structure prediction in the datasets we studied. (0.03 MB DOC) [file pgen.1000114.s006.pdf]

	CHORI & CORIELL	CHORI	CORIELL
45 NW/SE[1]	0.65	0.64	0.52
141 NW/SE & SE/AJ[1]	0.75	0.75	0.71
188 N/AJ[2]	0.89	0.89	0.89
377 N/AJ[2]	0.93	0.93	0.92
1419 N/AJ[2]	0.96	0.96	0.96

Table 1: **(supplementary)** Correlation coefficient between the "true" ancestry of our three European-American datasets and the predicted ancestry using five different sets of SNPs from Price et al.[1] and Tian et al.[2]. Tian et al.[2] report on sets of 1441, 384 and 192 N/AJ AIMs. However only 1419, 377 and 188 SNPs from each subset were present in the dataset we analyzed. It should also be noted that Price et al.[1] reported on 100 NW/SE and 300 NW/SE & SE/AJ markers, but only 45 and 141 markers respectively were available in our datasets, which might explain their somewhat inferior performance.

References

[1] Price A, Butler J, Patterson N, Capelli C, Pascali V, et al. (2008) Discerning the ancestry of European Americans in genetic association studies. PLoS Genet 4:e236.

[2] Tian C, Plenge R, Ransom M, Lee A, Villoslada P, et al. (2008) Analysis and application of European genetic substructure using 300 K SNP information. PLoS Genet 4:e4.
