# Supplementary material for: Tracing Sub-Structure in the European American Population with PCA-Informative Markers
Source: PLoS Genet. 2008 Jul 4;4(7):e1000114. doi: 10.1371/journal.pgen.1000114 (PMC2537989; doi:10.1371/journal.pgen.1000114)
Supplement: Table S2 — Overlap between European American AIMs proposed in studies of European American datasets. (0.03 MB PDF) [file pgen.1000114.s007.pdf]

References

[1] Price A, Butler J, Patterson N, Capelli C, Pascali V, et al. (2008) Discerning the ancestry of European Americans in genetic association studies. PLoS Genet 4:e236.

[2] Tian C, Plenge R, Ransom M, Lee A, Villoslada P, et al. (2008) Analysis and application of European genetic substructure using 300 K SNP information. PLoS Genet 4:e4.

	**(a)** 500 CHORI & CORIELL (this report)	**(b)** 500 CHORI (this report)	**(c)** 500 CORIELL (this report)	**(d)** 100 NW/SE (Price et al.[1])	**(e)** 300 NW/SE/AJ & SE/AJ (Price et al.[1])	**(f)** 192 N/AJ (Tian et al.[2])	**(g)** 384 N/AJ (Tian et al.[2])	**(h)** 1441 N/AJ (Tian et al.[2])
**(a)**		178	103	2	3	9	20	36
**(b)**			34	3	4	10	16	32
**(c)**				1	2	6	15	28
**(d)**					100	2	2	4
**(e)**						4	5	11
**(f)**							192	192
**(g)**								384

Table 1: **(supplementary)** Number of overlapping SNPs between eight sets of ancestry informative markers for European-American populations that appear in this paper and recent papers. The first three sets come from this paper and correspond to the top 500 PCAIMs for the CHORI dataset, the CORIELL dataset, and the joint dataset. The next two sets refer to the subsets of informative SNPs proposed in the Price et al.[1] paper and the last three SNP panels were proposed by Tian et al.[2]. It should be noted that the SNPs analyzed in each of these three studies were not the same. Price et al.[1] proposed 100 markers that were ancestry informative for the Northwest-Southeast Europe axis (**(d)** - only 45 of those SNPs were included in our analysis), as well as 300 markers that were ancestry informative for both the Northwest-Southeast axis and the Northern European-Ashkenazi Jewish axis (**(e)** - 141 of those SNPs were included in our analysis). On the other hand, Tian et al.[2] proposed panels of 192 AIMs ((**(f)**) - 188 were also included in our dataset), 384 (**(g)** - 377 SNPs included in our analysis), and 1,441 SNPs (**(h)** - 1,419 of these SNPs were present in the datasets we analyzed). We do not have information on the SNP overlap between the data analyzed by Price et al.[1] and Tian et al.[2]. However, it should be comparable to the overlap between our dataset and the Tian et al.[2] dataset (the same array was used for genotyping in our study and the Tian et al.[2] study).
