# Supplementary material for: Tracing Sub-Structure in the European American Population with PCA-Informative Markers
Source: PLoS Genet. 2008 Jul 4;4(7):e1000114. doi: 10.1371/journal.pgen.1000114 (PMC2537989; doi:10.1371/journal.pgen.1000114)
Supplement: Text S1 — Supplementary note. (0.06 MB PDF) [file pgen.1000114.s008.pdf]

Text S1 - Supplementary note

Simulation analysis

To demonstrate the performance of PCAIMs to correct for stratification in association studies on admixed populations with similar characteristics with European American populations, we followed the methods of [1] to generate an admixed population of 1,000 individuals genotyped on 100,000 SNPs. In order to generate the SNP genotypes, we first created 100,000 SNPs for two ancestral populations by creating random sets of allele frequencies between 0.05 and 0.95 via the Balding-Nichols model [1, 2] with $F_{st} = 0.01$. The latter number is typical of the differentiation between European populations [1, 3, 4]. Let f_i^1 and f_j^2 be the frequencies of the i-th SNP (for $i = 1 \ldots 100,000$) for the first and the second ancestral population respectively. Then, for each individual in the admixed population, we picked a random ancestry coefficient α_j, for $j = 1 \ldots 1,000$, and filled in genotypes for the i-th SNP of the j-th individual with respect to the frequencies $f_i^{adm} = \alpha_j \cdot f_i^1 + (1 - \alpha_j) \cdot f_i^2$. Thus, we created a $1,000 \times 100,000$ matrix A of genotypes.

In order to simulate an association study, we created large sets of random, stratified, and causal SNPs (100,000 SNPs in each case) following the methods described by Price et al. [1]. Random SNPs were created following the above methodology. Stratified SNPs (non-causal SNPs that nevertheless display a strong correlation with affection status), were simulated by allowing the ancestral allele frequencies to differ greatly between the two populations; our frequency choice was 0.8 for ancestral population 1, and 0.2 for ancestral population 2 as had been previously selected by Price et al [1]. Finally, causal SNPs were simulated by using a risk factor of $R = 1.5$ to modify the probability distribution of genotype values in causal SNPs as described in [1]. We performed ten repetitions, and generated sets of 100,000, since we did not observe any change in the fourth decimal digit of the reported results by increasing the set size to 1,000,000. Affection status for individuals in the admixed population was determined randomly according to an ancestry risk parameter r as defined previously [1]. The probability of the j-th individual being a case was set to $.5 * \log r \cdot \frac{r^{\alpha_j}}{r-1}$.

References

[1] Price A, Patterson N, Plenge R, Weinblatt M, Shadick N, et al. (2006) Principal components analysis corrects for stratification in genome-wide association studies . Nat Genet 38:904–909.

[2] Balding DJ, Nichols RA (1995) A method for quantifying differentiation between populations at multi-allelic loci and its implications for investigating identity and paternity. Genetica 96:3–12.

[3] Cavalli-Sforza LL, Menozzi P, Piazza A (1994) The History and Geography of Human Genes. Princeton, New Jersey: Princeton University Press, Princeton.

[4] Nicholson G, Smith AV, Jonsson F, Gustafsson O, Stefansson K, et al. (2002) Assessing population differentiation and isolation from single-nucleotide polymorphism data. JR Statist Soc 64:695–715.
